# Identification of sodium homeostasis genes in *Camelus bactrianus* by whole transcriptome sequencing

**DOI:** 10.1002/2211-5463.13380

**Published:** 2022-02-22

**Authors:** Dong Zhang, Jing Pan, Chunxia Liu, Fanhua Meng, Yanru Zhang, Junwei Cao, Yu Cao, Huanmin Zhou

**Affiliations:** ^1^ 117454 Inner Mongolia Key Laboratory of Bio‐manufacture Inner Mongolia Agricultural University Hohhot China; ^2^ 117454 College of Life Sciences Inner Mongolia Agricultural University Hohhot China; ^3^ Department of Reproductive Medicine Inner Mongolia Maternal and Child Health Care Hospital Hohhot China; ^4^ 58301 Institute of Traditional Chinese Medicine Tianjin University of Traditional Chinese Medicine Tianjin China

**Keywords:** camel, ceRNAs, renal medulla, salt resistance, sodium homeostasis

## Abstract

Salt dietary intake is tightly coupled to human health, and excessive sodium can cause strokes and cardiovascular diseases. Research into the renal medulla of camels exhibiting high salt resistance may aid identification of the mechanisms governing resistance to high salinity. In this study, we used RNA sequencing (RNA‐seq) to show that in the renal medulla of camels under salt stress, 22 mRNAs, 2 long noncoding RNAs (lncRNAs), and 31 microRNAs (miRNAs) exhibited differential expression compared with the free salt‐intake diet group. Using fluorescence *in situ* hybridization and dual‐luciferase reporter assays, we demonstrated that the lncRNA LNC003834 can bind miRNA‐34a and thereby relieve suppression of the salt‐absorption‐inhibiting *SLC14A1* mRNA from miRNA‐34a, suggesting that the above lncRNA‐miRNA‐mRNA act as competing endogenous RNAs (ceRNAs). We subsequently performed short hairpin RNA and small RNA interference and reactive oxygen species (ROS) detection assays to show that *SLC6A1*, *PCBP2,* and *PEX5L* can improve the antioxidation capacity of renal medulla cells of camel by decreasing ROS levels. Our data suggest that camels achieve sodium homeostasis through regulating the expression of salt‐reabsorption‐related genes in the renal medulla, and this involves ceRNAs (*SLC14A1* mRNA, LNC003834, and miRNA‐34a) and antioxidant genes (*SLC6A1*, *PCBP2,* and *PEX5L*). These data may assist in the development of treatments for diseases induced by high salt diets.

## Graphical abstract

The present study found that the lncRNA LNC003834 contributes to the upregulation of *SLC14A1* expression, which can prevent excessive sodium reabsorption via competitive binding with miRNA‐34a in the renal medulla of camel. The higher expression of *SLC6A1*, *PEX5L,* and *PCBP2* decreases reactive oxygen species levels to mitigate oxidative damage, contributing to the salt resistance exhibited by camels.

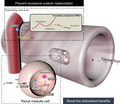

**Abbreviations**
ceRNAs: competing endogenous RNAs
CVDs: cardiovascular diseases
DHE: dihydroethidium
DMEM/F‐12: Dulbecco’s modified Eagle’s medium/nutrient mixture F‐12
FISH: fluorescence in situ hybridization
GABA: γ‐aminobutyric acid
lncRNAs: long noncoding RNAs
miRNAs: microRNAs
MUT: mutant type
NC: negative control
RNA‐seq: RNA sequencing
ROS: reactive oxygen species
sRNA: small RNA
WT: wild‐type

Salt is a mineral that is mainly composed of sodium chloride and normally exists in two natural forms including halite and sea salt. Sodium serves as an essential molecule in mammalian physiology for its roles as an electrolyte and osmotic solute [1, 2, 3]. The need to satisfy the sense of salty taste is innate and important [4], and the intake of an adult per day is less than 2000 mg of sodium (or 5 grams of salt) recommended by the World Health Organization [5]. But in many Western countries, the typical intake of salt is about 10 grams per day, which is higher than that in many countries in Asia and Eastern Europe [6]. Compared with Westerners, Asians are sensitive to salt and generally have higher blood pressure readings in the morning and at night [7]. According to guidelines by the United States, people with hypertension, middle‐aged, and older adults should control their intake to no more than 1500 mg of sodium per day [8]. High sodium in diets has been proved to be one of the most outstanding dietary risks [9]. Excessive dietary salt consumption will raise the risk of kidney damage, stroke, autoimmune diseases, including rheumatoid arthritis disease [10, 11], and cardiovascular diseases (CVDs) like hypertension [2, 6, 12, 13]. In addition, hypertonic sodium ion accumulation in peripheral tissues is associated with aging, hypertension, diabetes, chronic kidney disease, and heart failure [14]. In the United States and other westernized nations, CVDs usually trigger the death of adults over the age of 65 years old [15], while cutting salts from diet can lower blood pressure and decreases the risk of death from CVDs in people with hypertension [16, 17] and in adults and children with no acute illness [10, 17, 18, 19]. For instance, if sodium intake reduces to 1000 mg per day, the incidence of CVDs will decrease by about 30 percent [1].

Animals that live in the desert, such as the Gobi Desert, frequently confront the lack of food and tolerate high salinity water for a long period of time [20, 21]. Due to evolution and natural selection, camels (*Camelus bactrianus*) developed resistance to heat, drought, and salt for long‐term exposure in the desert [22]. The amount of daily salt needed to meet the requirements is 2 to 5 g in pigs as well as 3.9 g in goats [23, 24], for example. Regarding high salt consumption, camels should have 1.5 or 2 oz. (roughly 42.5 or 56.7 g) of salt daily or 4.5 to 6 oz. (approximately 127.6 to 170.1 g) of salt every three days [25]. As a salt‐metabolism‐related essential organ, the kidney primarily participates in the reabsorption of nutrients, waste filtration, and electrolyte homeostasis in mammals [26]. In the kidney, the renal medulla generally maintains the salt and water balance of the blood and consists of the vasa rectae (both spuria and vera), the venulae rectae, the medullary capillary plexus, the loop of Henle, and the collecting tubule [27, 28].

RNAs transcribed from DNAs are a class of vital molecules that have diverse biological roles. mRNAs take charge of directing protein synthesis and noncoding RNAs, such as miRNAs and lncRNAs, are involved in post‐transcriptional regulation [29, 30, 31, 32]. The studies including mammalian hibernation, frog and insect freeze tolerance, and turtle and marine snail anoxia tolerance have reported that the post‐transcriptional regulation mediated by noncoding RNAs is linked to biotic resistance under stress, for instance, that miRNA can rapidly halt the translation of unnecessary proteins [33]. Implicated in preventing sodium from entering the cell, the *SLC6A19* mRNA aimed by miR‐193b and miR‐542‐5p as well as the putative competing endogenous LNC001438, LNC003417, LNC001770, miR‐199c, and *TENM1* mRNA were considered as salt‐resisting modulators [34, 35].

Hence, taking transcriptome sequencing as a basis, our research targeting differential gene expression in the renal medulla of camels under salt stress will help scientists comprehending how the mammals achieve sodium homeostasis through natural instinct. The result of this study may offer an alternative investigation direction for solving the health problems due to excessive salt intake.

## Materials and methods

### Camels for salt stress treatment

The six female camels from 7.8 ± 0.4 years old (mean ± SD age) living in the Alxa Gobi Desert of northern China were penned and randomly divided into two groups (three camels per group): The experimental group was under salt stress condition, and the control group was under free salt‐intake diet, both groups were assessed for the consecutive periods of 24 days. The mean weight of the six camels was 352.6 ± 7.1 kg (Table S1). In the salt stress group, the extra salt intake base was 200 g·day^−1^ and increased by 100 g every 3 days, and the daily intake of salt was 900 g at the end of the experiment, following free water and feeding. Camels ate and drank freely in control group, with daily 50 g salt consumption. Procedures involving animals, their care, and the humane kill were conducted in conformity with the Guidelines on the Humane Treatment of Laboratory Animals (HTLA Pub. Chapter 2‐6, revised 2006 in China) and were approved by the Animal Care and Use Committee of the Inner Mongolia Agricultural University (IMAU‐IACUC‐2019‐31560313). The written informed consent of the experimental research was achieved by S. Wang (as an animal owner).

### Tissue sample collection

After injecting 0.5 mg·kg^−1^ xylazine (Table S1), the six Alxa bactrian camels of the two groups were sacrificed by bleeding of the carotid artery. Renal medulla tissues for the study were sampled and stored in 1.5 mL frozen tubes and then immediately deposited in liquid nitrogen (−196 °C) for preservation and continuous experiments.

### RNA library construction and bioinformatics analysis

Total RNAs were extracted from renal medulla tissues of camels using the Qiagen RNeasy Mini Kit (QIAGEN, Germany). Potential RNA degradation and contamination were checked on 1% agarose gels. The purity, concentration, and integrity of total RNAs were determined by the NanoPhotometer spectrophotometer (IMPLEN, USA), the Qubit RNA Assay Kit in Qubit 2.0 Fluorometer (Life Technologies, USA), and the RNA Nano 6000 Assay Kit of the Agilent Bioanalyzer 2100 system (Agilent Technologies, USA), respectively. Three micrograms of total RNA per sample were utilized for small RNA and lncRNA library construction. The sequencing libraries of small RNA and lncRNA were individually created using the NEBNext Multiplex Small RNA Library Prep Set for Illumina (NEB, USA) and the NEBNext Ultra Directional RNA Library Prep Kit for Illumina (NEB, USA). These steps were preceded by the removal and cleaning up of ribosomal RNA using the Epicentre Ribo‐zero rRNA Removal Kit (Epicentre, USA) and ethanol precipitation. Pooled RNA‐seq was applied to the constructed libraries of small RNA and lncRNA using an Illumina HiSeq 2500 platform and 4000 platform in turn [36]. The miRNA data were analyzed by quality control, reads mapping to *Camelus bactrianus* genome (NCBI reference genome: Ca_bactrianus_MBC_1.0; RefSeq: GCF_000767855.1) using Bowtie (parameters: ‐v 0 ‐k 1) [37], and novel miRNA prediction with miREvo (parameters: ‐i ‐r ‐M ‐m ‐k ‐p 10 ‐g 50000) [38] and mirdeep2 (parameters: quantifier.pl ‐p ‐m ‐r ‐y ‐g 0 ‐T 10) [39]. The differential expression of miRNA was analyzed via TPM [40] and DEGseq with qvalue < 0.01 and |log2(foldchange)| > 1 [41]. Small RNA annotation was investigated using the *Bos taurus* database. The prediction of target genes was performed via miRanda (parameters: ‐sc 140 ‐en −10 ‐scale 4 ‐strict ‐out) and RegRNA2.0 (parameters: score ≥ 170; free energy ≤ −15). The lncRNA data were analyzed by quality control, reads mapping to *Camelus bactrianus* genome (NCBI reference genome: Ca_bactrianus_MBC_1.0; RefSeq: GCF_000767855.1) using TopHat v2.0.9 (parameters: ‐‐library‐type fr‐firststrand) [42] and Cufflinks (parameters: min‐frags‐per‐transfrag = 0) transcript assembly [43]. lncRNA and mRNA were sorted by CNCI v2 with default parameters [44], CPC 0.9‐r2 (parameters: ‐evalue 1e‐10) [45], Pfam Scan v1.3 (parameters: ‐E 0.001 ‐‐domE 0.001 ‐pfamB) [46] and PhyloCSF v20121028 (parameters: ‐‐orf = ATGStop; ‐frames = 3; ‐removeRefGaps) [47]. Annotations of mRNA and lncRNA were accomplished using *Camelus bactrianus* and *Bos taurus* databases, and the differential expression analysis of lncRNA and mRNA was exerted by Cuffdiff (http://cole‐trapnell‐lab.github.io/cufflinks/cuffdiff/index.html). The prediction of target gene was carried out by lncRNA gene upstream/downstream 100 kb and Pearson correlation coefficient with |Pearson correlation| > 0.95 [48]. Gene Ontology and Kyoto Encyclopedia of Genes and Genomes pathway enrichment analyses of target genes and differentially expressed mRNAs were conducted by GOseq [49] and KOBAS 2.0 (parameters: blastx 1e‐10 and padjust: BH) [50].

### RNA fluorescence in situ hybridization (FISH) of camel renal medulla tissues

FISH was performed on renal medulla tissues from salt stress and control camels in accordance with the protocol of Fluorescence *in situ* Hybridization Kit (GEFAN, China) through fluorescent groups (FAM and Cy3) labeled RNA probes and DAPI stain. The sequences of fluorescent group labeled RNA probes are listed in Table S2.

### Cell isolation and cultivation

The sampled renal medullas of deceased camel were removed and cleansed by Dulbecco’s phosphate‐buffered saline (Solarbio, China) with 1% penicillin and 1% streptomycin (NCPC, China). Following chopping, the small pieces of renal medulla tissue were seeded on the T25 culture bottle (Corning, Germany) and cultivated with Dulbecco’s modified Eagle’s medium/nutrient mixture F‐12 (DMEM/F‐12) (Hyclone, USA) containing 10% fetal bovine serum (Sigma, USA), 0.1% penicillin, and 0.1% streptomycin (NCPC, China) in a humidified chamber with 5% CO_2_ at 37 °C. The cells migrated out around tissues were initiated into digestion via using trypsin‐EDTA solution (Solarbio, China) at a cell growth confluence of 80%. The renal medulla cells of camels those in good growth condition were selected for the subsequent experiments after passage. Besides renal medulla cells, the 293T cells were also cultured in the above‐modified DMEM/F‐12 with 5% CO_2_ at 37 °C.

### Dual‐luciferase reporter assay

The formula of high transfection efficiency was explored in 293T cells, using NC‐FAM oligo and pcDNA3.1‐GFP plasmid (GenePharma, China). Different co‐transfected combinations, miRNA‐34a oligo (GenePharma), NC oligo (GenePharma), LNC003834 plasmid vector including wild‐type (WT) and mutant type (MUT) (LNC003834S1 WT, LNC003834S2 WT, LNC003834S1 MUT, and LNC003834S2 MUT, GenePharma), SLC14A1 luciferase reporter plasmid WT and MUT (SLC14A1 WT and SLC14A1 MUT, GenePharma) were transfected into the 293T cells by lipofectamine 2000 with a 70% transfection efficiency in line with the proportion of fluorescent cells. The cells were lysed after 48 h and the fluorescent intensity of luciferase was detected using Dual‐Luciferase Reporter Assay System (Promega, USA). The sequences of the corresponding RNAs are listed in Table S3 and Table S4. The functional sequences of LNC003834S1 and LNC003834S2 were as follows:

a. LNC003834S1 WT: 5’‐AGGAACATAGGGACACTGCCC‐3’,

b. LNC003834S2 WT: 5’‐TTGTCATTCTTTTGCACTGCCC‐3’,

c. LNC003834S1 MUT: 5’‐TCCTTGTATCCCTGTGACGGG‐3’,

d. LNC003834S2 MUT: 5’‐AACAGTAAGAAAACGTGACGGG‐3’.

### Short hairpin RNA and small RNA interference

For shRNA‐ and sRNA‐mediated *SLC6A1*, *PEX5L,* and *PCBP2* suppression, the packaging shRNA lentivectors and small RNA oligos with attached GP‐siRNA‐Mate Plus reagent were purchased from GenePharma (China). 48 h after the transfection into camel renal medulla cells, the fluorescent intensity was detected according to the manufacturer’s protocol. The synthesized shRNA and sRNA sequences are listed in Table S5 and Table S6.

### RNA isolation and qRT‐PCR

RNAs were extracted from renal medulla tissues and cells of camel using mirVana miRNA Isolation Kit (Ambion, USA). The primers were designed by Primer Express 3.0.1 (Table S7) and qRT‐PCRs were exerted on the 7900 HT Sequence Detection System (ABI, USA).

### Western blot

The proteins were extracted by M‐PER Mammalian Protein Extraction Reagent (Thermo, USA). SLC6A1, PCBP2, and PEX5L protein expression were severally examined using antibodies against‐SLC6A1 (ab426 and ab72448, Abcam, UK) and PCBP2 (ab96169, Abcam, UK) and PEX5L (nbp1‐91484, Novus Biologicals, USA). The signals were monitored via Pierce ECL Western Blotting Substrate (Sangon Biotech, China).

### Reactive oxygen species detection

ROS staining was arranged after RNA interference in renal medulla cells by ROS fluorescence probe‐DHE (KeyGEN, China). The red fluorescence levels were analyzed by BD FACSDiva 8.0.1.1 on BD FACS flow cytometry (BD Biosciences, USA).

### Statistical analysis

Aside from RNA‐seq data statistics based on negative binomial distribution, Student’s t‐test was used to compare the mean values between groups and a P‐value of less than 0.05 was considered as statistically significant.

## Results

### Differentially expressed genes in renal medulla under salt load

**Fig. 1:**
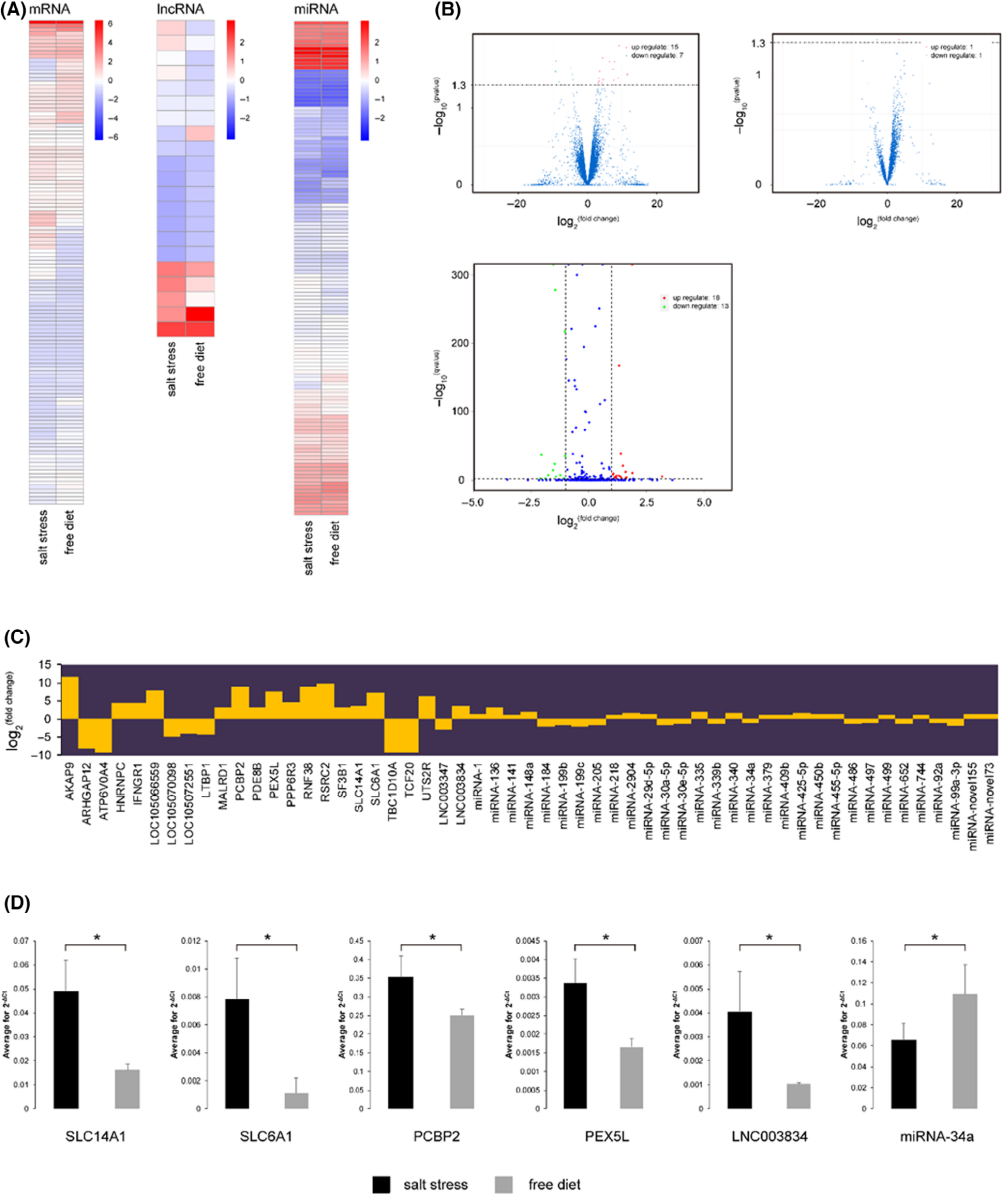
Differentially expressed genes in renal medulla of camels subjected to salt stress or free diet. (A) Differential log_10_^(FPKM+1)^ values of mRNA and lncRNA and log_10_^(TPM+1)^ values of miRNA. (B) Number and (C) log_2_^(fold change)^ value of significantly differential mRNA, lncRNA, and miRNA genes. (D) Average 2^‐ΔCt^ value of genes assessed by qRT‐PCR. Data are presented as mean ± SD (*n* = 3 camels) by Student’s t‐test. Symbols: *P < 0.05. β‐actin and U6 were used as loading control for mRNA and lncRNA, and miRNA, respectively.

RNA‐seq analysis identified 22 mRNAs (15 upregulated and 7 downregulated), 2 lncRNAs (1 upregulated and 1 downregulated), and 31 miRNAs (18 upregulated and 13 downregulated) that were differentially (*P* < 0.05) expressed in camel renal medulla by comparing high‐salt group and control group (Fig. 1A and B; Table S8 and Table S9). We noticed that *SLC14A1*, a molecule involved in salt absorption [51, 52], displayed significantly higher expression intensity grade in salt stress (Fig. 1C). Other upregulated molecules, such as *SLC6A1*, *PEX5L,* and *PCBP2* (Fig. 1C), are speculated as prospective mediators against reactive oxygen species (ROS) [53, 54, 55, 56, 57]. Moreover, *SLC14A1* mRNA and a novel lncRNA LNC003834 (XLOC_182366, NW_011544901.1) were identified as underlying targets of downregulated miRNA‐34a, suggesting that *SLC14A1* mRNA, LNC003834, and miRNA‐34a could be competing endogenous RNAs (ceRNAs). The ceRNAs function has been clarified in regulating RNA transcripts via competing for shared miRNAs at the post‐transcriptional level [58, 59]. Therefore, the above 4 protein‐coding genes (*SLC14A1*, *SLC6A1*, *PEX5L*, and *PCBP2*) and the 2 noncoding genes (LNC003834 and miRNA‐34a) were screened out as candidate genes of salt resistance in camel renal medulla. When considering qRT‐PCR results, their different levels were consistent with RNA‐seq gene expression analyses (Fig. 1D).

### Colocated LNC003834 and miRNA‐34a in extranuclear region

**Fig. 2:**
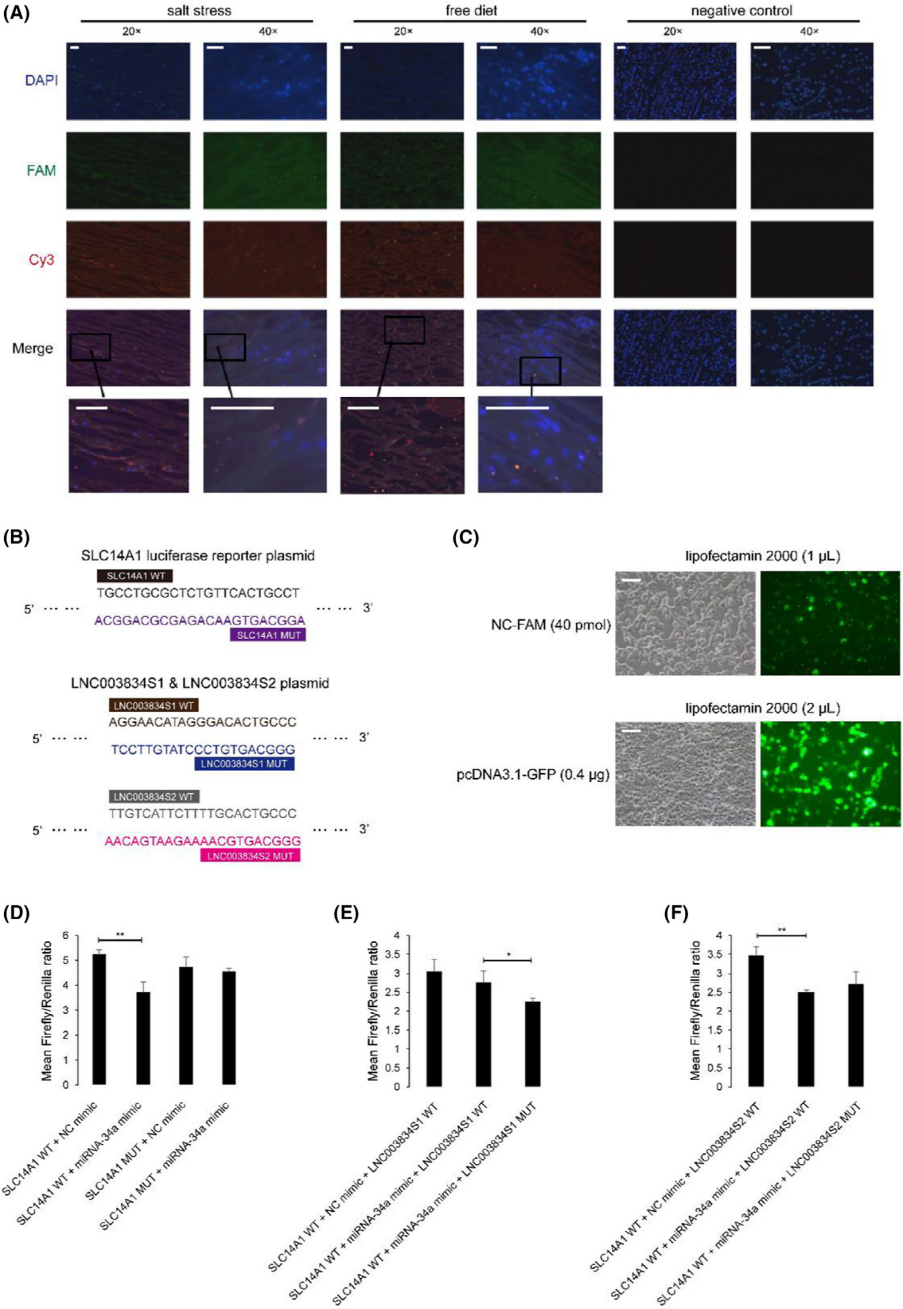
Competing endogenous *SLC14A1* mRNA, miRNA‐34a, and LNC003834. (A) Immunofluorescence microscopy image of camel renal medulla under salt stress and free diet (20× and 40×). LNC003834, miRNA‐34a, and nucleus were independently labeled by FAM (green), Cy3 (red), and DAPI (blue). Scale bar, 500 μm. (B) The inserted sequence in SLC14A1 luciferase reporter plasmid and LNC003834 (LNC003834S1 and LNC003834S2) plasmids including two potential binding regions. (C) The experimental conditions with 70% transfection efficiency of NC‐FAM oligo and pcDNA3.1‐GFP plasmid (green) in 293T cells *in vitro* (200×). Scale bar, 100 μm. (D–F) Mean Firefly/Renilla ratio of miRNA‐34a mimic, SLC14A1 WT and SLC14A1 MUT, LNC003834S1 WT and LNC003834S1 MUT, and LNC003834S2 WT, and LNC003834S2 MUT. WT and MUT separately refer to wild‐type and mutant type. Data are presented as mean ± SD (*n* = 3) by Student’s *t*‐test. Symbols: *P < 0.05; **P < 0.01.

Using FISH techniques, the cellular localizations of LNC003834 and miRNA‐34a were tracked in camel renal medulla tissues. FAM fluorescent group‐labeled lncRNA and Cy3 fluorescent group‐conjugated miRNA probes were designed and synthesized according to the sequences of LNC003834 (Table S10) and miRNA‐34a (miRBase, Accession: MIMAT0004340, ID: bta‐miR‐34a). By fluorescence microscopy, LNC003834 (green) and miRNA‐34a (red) presented nonoverlapping fluorescence against DAPI‐stained nuclei (blue) (Fig. 2A). The results signify that the localization of potential interaction between LNC003834 and miRNA‐34a is extranuclear.

### Competing endogenous LNC003834, miRNA‐34a, and SLC14A1

In the dual‐luciferase reporter assay, SLC14A1 wild‐type and mutant type luciferase reporter plasmids (SLC14A1 WT and SLC14A1 MUT), which contain original or base‐altered for miRNA‐34a potential binding sequence (SLC14A1 WT: 5’‐TGCCTGCGCTCTGTTCACTGCCT‐3’, SLC14A1 MUT: 5’‐ACGGACGCGAGACAAGTGACGGA‐3’) (Fig. 2B), were transfected into 293T cells by liposome. The synthetic miRNA‐34a (miRBase, Accession: MIMAT0004340, ID: bta‐miR‐34a) oligo, LNC003834 WT and MUT plasmids (LNC003834S1 WT, LNC003834S2 WT, LNC003834S1 MUT, and LNC003834S2 MUT), including the material or base‐changed the miRNA‐34a potential binding sequences (Fig. 2B), were also transfected into 293T cells. A 70% transfection efficiency of NC‐FAM oligo and pcDNA3.1‐GFP plasmid was obtained with 40 pmol oligo with 1 L lipofectamine 2000 and 0.4 g plasmid with 2 L lipofectamine 2000 (Fig. 2C). The test results indicated that the average value of the Firefly/Renilla luciferase ratio of the ‘SLC14A1 WT + miRNA‐34a mimic’ group was significantly (*P* < 0.01) lower than the ‘SLC14A1 WT + negative control (NC) mimic’ group (Fig. 2D). However, the Firefly/Renilla ratio of the ‘SLC14A1 MUT + miRNA‐34a mimic’ group and the ‘SLC14A1 MUT + NC mimic’ group were not statistically significant (Fig. 2D). The results suggest that miRNA‐34a inhibits *SLC14A1* expression by its binding to target sequence on *SLC14A1* mRNA. The ‘SLC14A1 WT + miRNA‐34a mimic + LNC003834S1 WT’ group showed no significant difference with the Firefly/Renilla ratio when compared with the ‘SLC14A1 WT + NC mimic + LNC003834S1 WT’ group and markedly (*P* < 0.05) higher Firefly/Renilla ratio value than the ‘SLC14A1 WT + miRNA‐34a mimic + LNC003834S1 MUT’ group (Fig. 2E). In terms of the prospective LNC003834S2, the ‘SLC14A1 WT + miRNA‐34a mimic+ LNC003834S2 WT’ group exhibited significantly (*P* < 0.01) lower Firefly/Renilla ratio than the ‘SLC14A1 WT + NC mimic + LNC003834S2 WT’ group, while there was no apparent difference of Firefly/Renilla ratio between the ‘SLC14A1 WT + miRNA‐34a mimic + LNC003834S2 WT’ group and the ‘SLC14A1 WT + miRNA‐34a mimic + LNC003834S2 MUT’ group (Fig. 2F). The results demonstrate that LNC003834S1 reduces the inhibition of *SLC14A1* expression by emulous binding miRNA‐34a, but not LNC003834S2. Generally, these results supply evidence that miRNA‐34a can target and bind the seed region of *SLC14A1* mRNA and depress *SLC14A1* expression, while LNC003834S1 weakens the inhibitory effect by competitively binding miRNA‐34a.

### Establishment of stable SLC6A1, PEX5L, and PCBP2 short hairpin RNAs (shRNAs) expressing camel renal medulla cell lines

**Fig. 3:**
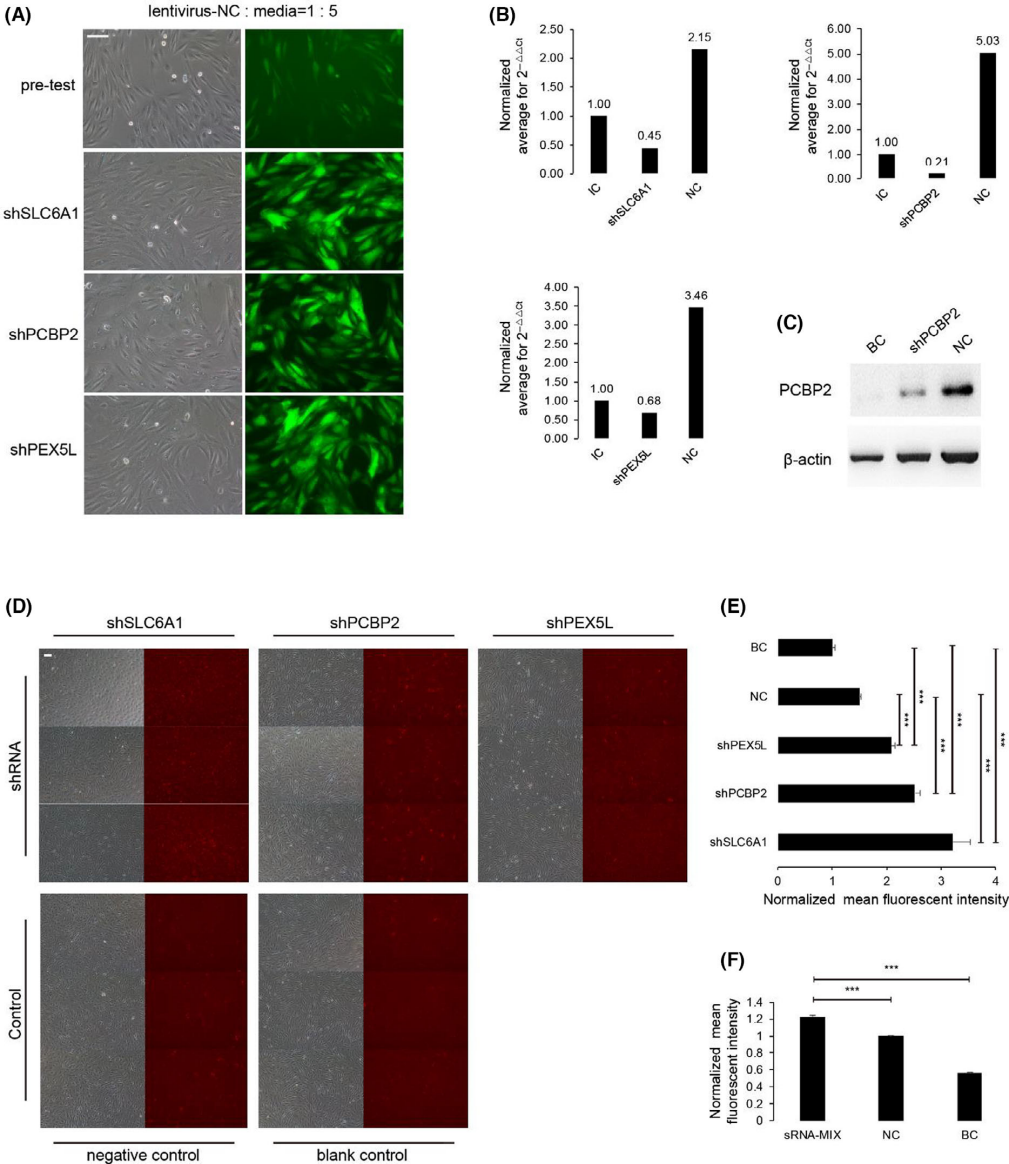
RNA interference and ROS detection. (A) The experimental formulas of achieved 70% transfection efficiency of shRNA lentiviral vectors targeting *SLC6A1*, *PCBP2,* and *PEX5L* in renal medulla cells of camel *in vitro* with lentivirus‐NC/media = 1/5 (200×). Scale bar, 100 μm. (B) Expression levels of *SLC6A1*, *PCBP2,* and *PEX5L* in the light of the ΔΔCt method in different groups. GAPDH was used as a loading control (intrinsic control), and expression levels were normalized with *SLC6A1*, *PCBP2,* and *PEX5L* mRNA levels of NC and RNA interference groups. (C) Western blot targeting PCBP2. β‐actin was used as a loading control. (D) ROS detection after shRNA interference in renal medulla cells of camel by red fluorescent imaging DHE probe (100×). Scale bar, 100 μm. (E,F) Fluorescence intensity of ROS after shRNA interference against *SLC6A1*, *PCBP2,* and *PEX5L*, and after small RNA interference against mixed *SLC6A1*, *PCBP2,* and *PEX5L* (sRNA‐MIX). Data are presented as mean ± SD (*n* = 3) by Student’s t‐test. Symbols: ***P < 0.001. shSLC6A1, shPCBP2, and shPEX5L represent short hairpin RNAs of SLC6A1, PEX5L, and PCBP2 in turn. IC refers to intrinsic control with GAPDH, NC indicates negative control with nonsense shRNA, and BC means blank control.

Based on the sequences of *SLC6A1*, *PEX5L,* and *PCBP2*, the corresponding shRNAs were designed and synthesized. In line with 70% transfection efficiency (lentivirus fluid/cell media = 1/5, total volume = 1 mL) (Fig. 3A), shRNAs against *SLC6A1*, *PEX5L,* and *PCBP2* were transfected into cultured renal medulla cells of camel by lentivirus packaging. The qRT‐PCR results showed that the expression levels of *SLC6A1*, *PEX5L,* and *PCBP2* were decreased following transfections (Fig. 3B), representing that the expression of *SLC6A1*, *PEX5L,* and *PCBP2* can be inhibited effectively by lentivirus‐mediated shRNAs expression. Regretfully, western blot results only detected PCBP2 protein expression (Fig. 3C), SLC6A1 and PEX5L had no clear bands. The possible main reason is that the SLC6A1‐ and PEX5L‐antibodies are not suitable for detecting the two proteins in camel cells with the absence of specific antibodies.

### Surging ROS levels in SLC6A1, PEX5L, and PCBP2 shRNAs and small RNA (sRNA)‐MIX renal medulla cells

The ROS levels were detected by dihydroethidium (DHE) fluorescent probe in renal medulla cells. Fluorescence imaging and intensity reflected that ROS levels were significantly higher (*P* < 0.001) in *SLC6A1*, *PEX5L,* and *PCBP2* shRNAs and sRNA‐MIX cells (three oligos of *SLC6A1*, *PEX5L* and *PCBP2* small RNA co‐interference) when compared with the negative and blank controls (Fig. 3D–F; Fig. S1). The results manifest that defective expression of *SLC6A1*, *PEX5L,* and *PCBP2* cause increased ROS levels in camel renal medulla cells.

## Discussion

In the previous work of our laboratory, the camel’s complex features coupled with desert adaptations such as water and fat metabolism had been unveiled through comparative genomic analysis, as well as the unique osmoregulation, osmoprotection, and compensatory mechanisms for water reservation responding to aridity stress based on transcriptomic evidences [60]. This study detected 22 mRNAs, 2 lncRNAs, and 31 miRNAs that were differentially expressed in renal medulla tissues of camels subjected to salt stress or free diet by RNA‐seq. In the identified protein‐coding genes, *SLC14A1*, *SLC6A1*, *PEX5L,* and *PCBP2* were predicted to positively respond to salt stress, and *SLC14A1* mRNA may compete for endogenous RNA with a novel lncRNA LNC003834 and miRNA‐34a. According to a former research [61], *SLC14A1* encodes for the urea transporter B and is expressed in endothelial cells of the descending vasa recta of mammalian kidneys. In the renal medulla, the urea transporter B can improve urinary concentration ability and water reabsorption via its urea transmembrane transporter activity, and the single channel water permeability is similar to that of aquaporin 1 [62, 63]. In comparison to Dahl salt‐resistant rats or Dahl salt‐sensitive rats with normal salt diet, the *SLC14A1* mRNA levels were appreciably reduced in the choroid plexus of Dahl salt‐sensitive rats on high salt diet, indicating that reduced *SLC14A1* expression was correlated to increased sodium ion levels in the cerebrospinal fluid and elevated average arterial pressure [52]. Additionally, based on prior studies, *SLC6A1* is involved in sodium‐ and chloride‐dependent γ‐aminobutyric acid (GABA) transportation [53, 64]. GABA can influence salt tolerance of Arabidopsis and actively responds to heat stress in four‐year‐rice and salt stress in maize plumule by strengthening antioxidant ability [54, 65], whereas rare are animal studies that explored the relationship between GABA and environmental stress resistance. *PCBP2*‐deficient mice and primary mouse embryonic fibroblasts emerge ROS production increment and cellular senescence acceleration [55]. *PEX5L* is engaged in the peroxisome matrix targeting signal‐1 binding and the peroxisome targeting sequence binding in which the peroxisome dysfunction is associated with fatal oxidative damage [56, 66, 67]. Furthermore, we verified the expression levels of candidate salt‐resistance‐related molecules including *SLC14A1*, *SLC6A1*, *PEX5L*, *PCBP2*, LNC003834, and miRNA‐34a to be consistent with RNA‐seq outcomes by qRT‐PCR.

For ceRNAs studies which contained *SLC14A1* mRNA, LNC003834, and miRNA‐34a, several experiments were performed. The fluorescence distribution of the FISH images appeared that the functional areas of LNC003834 and miRNA‐34a were localized in the extranuclear space of the camel renal medulla. Relying on dual‐luciferase reporter assay system, we subsequently found that LNC003834 can act as a molecular sponge for miRNA‐34a to regulate *SLC14A1* expression and presented that: (a) *SLC14A1* expression was evidently restrained by miRNA‐34a due to the densely bound 23 seed matches of 3’ tail sequence of *SLC14A1* mRNA; (b) The newly recognized 21 seed matches of LNC003834 can reverse the miRNA‐34a‐mediated *SLC14A1* suppressed expression via competitively binding miRNA‐34a. These experiment results demonstrated relationships between LNC003834, miRNA‐34a, and *SLC14A1* mRNA and that were identified as competing endogenous RNAs with maintaining sodium homeostasis.

**Fig. 4:**
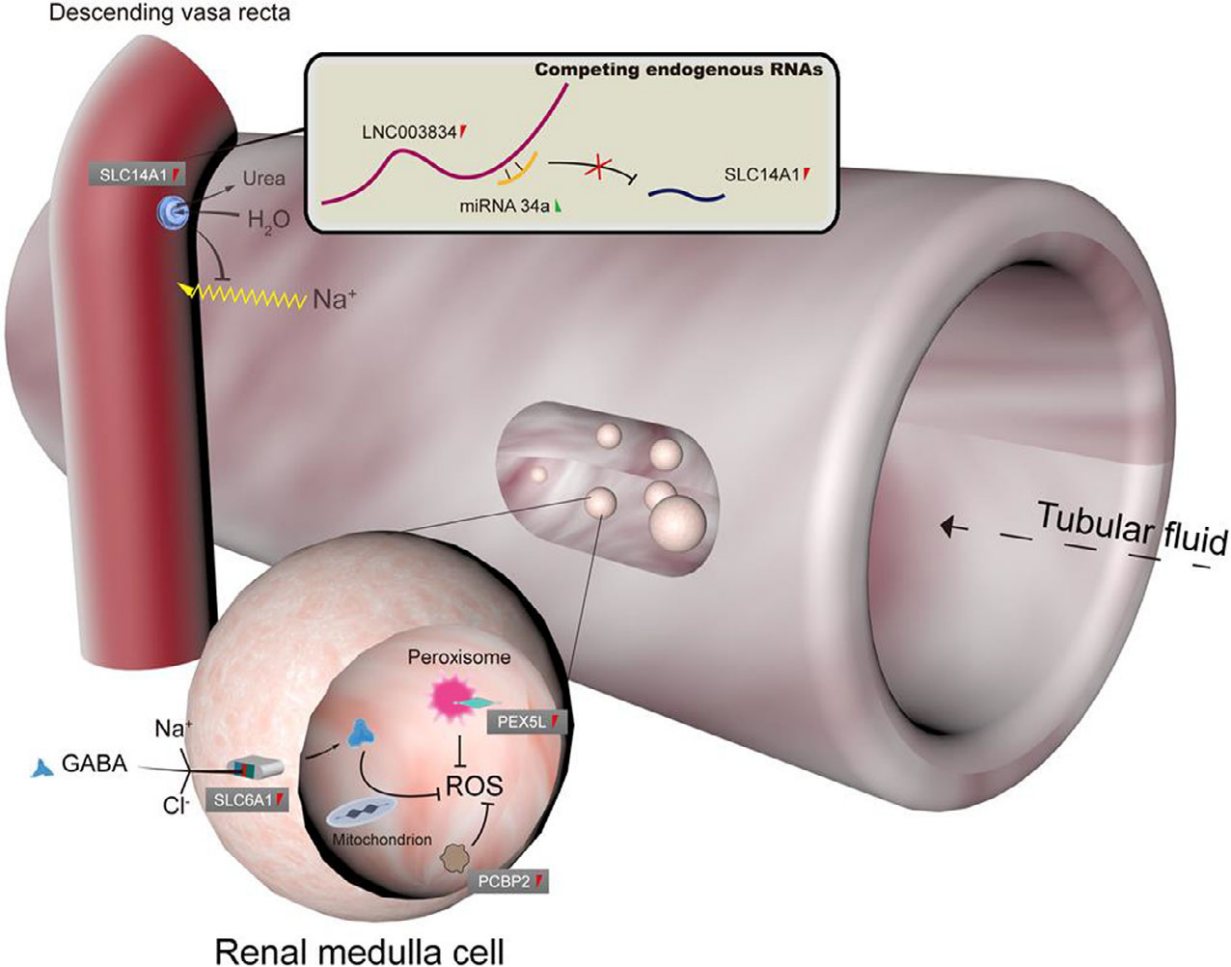
Salt‐resistance metabolism combined with competing endogenous RNAs (LNC003834, miRNA‐34a, and SLC14A1) and transcribed antioxidant genes (*SLC6A1*, *PCBP2,* and *PEX5L*) in renal medulla of camel.

At present, the causes of salt sensitivity can be mainly explained by two theories [68]. In the historical natriuretic handicap theory, salt sensitivity originates from a renal functional impairment, which represents the phenomena of slow excretion and sodium retention. In the contemporary vasodysfunction theory, salt‐induced hypertension results from abnormal vascular resistance responses under high salt intake. Notably in this research of biological salt resistance, we discovered a critical protein‐coding gene which contributes to enhancing vascular resistance in renal medulla with high salt consumption. The upregulated expression of *SLC14A1* may inhibit sodium ion reabsorption from the renal tube to the descending vasa recta through competing endogenous LNC003834, miRNA‐34a, and *SLC14A1* mRNA in camel renal medulla (Fig. 4). The examination of plasma Na^+^ concentration provided further evidence that no statistical significance was attained by comparing salt stress group and control group (Table S11).

Following shRNA knockdown of *SLC6A1*, *PEX5L* and *PCBP2*, and sRNA‐MIX interference, we learned that ROS levels were palpably increased in the renal medulla cells of camel. The occurrences meant that upregulated expression of *SLC6A1*, *PEX5L,* and *PCBP2* may ameliorate antioxidative effects in positive response to salt resistance (Fig. 4). It also hints that camel as one of mammal species can invoke *SLC6A1* gene to meet effortlessly to environmental challenges like plants do.

In summary, the present work exhibits that the inhibition of sodium reabsorption is activated by stronger *SLC14A1* expression which induced by ceRNAs comprising LNC003834 and miRNA‐34a; while, *SLC6A1*, *PEX5L,* and *PCBP2* reduce ROS levels via increased expression to achieve salt resistance in renal medulla of camel. It indicates that although with high dietary intake of salt, the reabsorption of sodium ion is inhibited. Herein, we reveal a mechanism for maintaining sodium homeostasis in camel and envision that our work can empower people to seek out a new way for solving human diseases triggered by high salt intake.

## Conflict of interest

The authors declare that there is no conflict of interest regarding the publication of this article.

## Author contributions

DZ conceived and designed the study. DZ, JP, CL, FM, and YC carried out the experiments. YC analyzed the data and wrote the manuscript. YZ, JC, and HZ provided valuable insights in the discussion and revision of the manuscript. All authors read and approved the final manuscript.

## Supporting information


**Fig. S1**. ROS detection after sRNA‐MIX interference in renal medulla cells of camel by red fluorescent imaging DHE probe (100×). sRNA‐MIX indicates three classes of small RNAs (sSLC6A1, sPEX5L and sPCBP2) co‐interference against *SLC6A1*, *PEX5L* and *PCBP2*. Scale bar, 100 μm.
**Table S1**. The details of six camels.
**Table S2**. The sequences of fluorescent group labeled RNA probes.
**Table S3**. The inserted SLC14A1 sequences in SLC14A1 luciferase reporter plasmids.
**Table S4**. The inserted LNC003834 sequences in LNC003834S1 and LNC003834S2 plasmids.
**Table S5**. The sequences of different short hairpin RNAs (shRNAs).
**Table S6**. The sequences of different small RNAs (sRNAs).
**Table S7**. The primer sequence of candidate genes of salt‐resistance.
**Table S8**. The significantly differential mRNAs and lncRNAs in the renal medulla of camel under salt stress.
**Table S9**. The significantly differential miRNAs in the renal medulla of camel under salt stress.
**Table S10**. The sequence of novel lncRNA LNC003834 gene.
**Table S11**. Plasma Na^+^ concentration.

## Data Availability

The RNA‐seq data from this study were deposited in NCBI Sequence Read Archive under accession SRA: SRP355729. The data screened and analyzed during this study were included in this article.

## References

[1] McGuire S . Scientific Report of the 2015 Dietary Guidelines Advisory Committee. Washington, DC: US Departments of Agriculture and Health and Human Services, 2015. Adv Nutr. 2016;7:202–4. 26773024 10.3945/an.115.011684 PMC4717899

[2] Strom BL , Yaktine AL , Oria M . Sodium Intake in Populations: Assessment of Evidence. Washington, USA: National Academies Press; 2013. 24851297

[3] Sasidhar V , Ruckenstein E . Electrolyte osmosis through capillaries. J Colloid Interf Sci. 1981;82:439–57.

[4] Rabinerson D , Horovitz E , Beloosesky Y . The sense of taste. Harefuah. 2006;145(601–605):629. 16983846

[5] Härtl G . WHO issues new guidance on dietary salt and potassium. Cent Eur J Public Health. 2013;21:16. 23741892

[6] Strazzullo P , D’Elia L , Kandala NB , Cappuccio FP . Salt intake, stroke, and cardiovascular disease: meta‐analysis of prospective studies. BMJ. 2009;339:b4567. 19934192 10.1136/bmj.b4567 PMC2782060

[7] Sogunuru GP , Kario K , Shin J , Chen CH , Buranakitjaroen P , Chia YC , et al. Morning surge in blood pressure and blood pressure variability in Asia: Evidence and statement from the HOPE Asia Network. J Clin Hypertens. 2019;21:324–34. 10.1111/jch.13451 PMC8030409 30525279

[8] McGuire S . U.S. Department of Agriculture and U.S. Department of Health and Human Services, Dietary Guidelines for Americans, 2010. 7th Edition, Washington, DC: U.S. Government Printing Office, January 2011. Adv Nutr. 2011;2:293–4. 22332062 10.3945/an.111.000430 PMC3090168

[9] GBD . Risk Factor Collaborators (2018) Global, regional, and national comparative risk assessment of 84 behavioural, environmental and occupational, and metabolic risks or clusters of risks for 195 countries and territories, 1990–2017: a systematic analysis for the Global Burden of Disease Study 2017. Lancet. 2017;392:1923–94. 10.1016/S0140-6736(18)32225-6 PMC6227755 30496105

[10] Sharif K , Amital H , Shoenfeld Y . The role of dietary sodium in autoimmune diseases: the salty truth. Autoimmun Rev. 2018;17:1069–73. 30213699 10.1016/j.autrev.2018.05.007

[11] Haase S , Wilck N , Kleinewietfeld M , Müller DN , Linker RA . Sodium chloride triggers Th17 mediated autoimmunity. J Neuroimmunol. 2019;329:9–13. 29983198 10.1016/j.jneuroim.2018.06.016

[12] He FJ , Li J , Macgregor GA . Effect of longer term modest salt reduction on blood pressure: cochrane systematic review and meta‐analysis of randomised trials. BMJ. 2013;346:f1325. 23558162 10.1136/bmj.f1325

[13] Xu H , Qing T , Shen Y , Huang J , Liu Y , Li J , et al. RNA‐seq analyses the effect of high‐salt diet in hypertension. Gene. 2018;677:245–50. 30059752 10.1016/j.gene.2018.07.069

[14] Rossitto G , Touyz RM , Petrie MC , Delles C . Much Ado about N…atrium: modelling tissue sodium as a highly sensitive marker of subclinical and localized oedema. Clin Sci (Lond). 2018;132:2609–13. 30545897 10.1042/CS20180575 PMC6365627

[15] Liu Y , Bloom SI , Donato AJ . The role of senescence, telomere dysfunction and shelterin in vascular aging. Microcirculation. 2019;26:e12487. 29924435 10.1111/micc.12487 PMC7135943

[16] Graudal NA , Hubeck‐Graudal T , Jurgens G . Effects of low sodium diet versus high sodium diet on blood pressure, renin, aldosterone, catecholamines, cholesterol, and triglyceride. Cochrane Database Syst Rev. 2020;12:CD004022. 33314019 10.1002/14651858.CD004022.pub5 PMC8094404

[17] Suckling RJ , Swift PA . The health impacts of dietary sodium and a low‐salt diet. Clin Med (Lond). 2015;15:585–8. 26621954 10.7861/clinmedicine.15-6-585 PMC4953267

[18] Aburto NJ , Ziolkovska A , Hooper L , Elliott P , Cappuccio FP , Meerpohl JJ . Effect of lower sodium intake on health: systematic review and meta‐analyses. BMJ. 2013;346:f1326. 23558163 10.1136/bmj.f1326 PMC4816261

[19] He FJ , MacGregor GA . Role of salt intake in prevention of cardiovascular disease: controversies and challenges. Nat Rev Cardiol. 2018;15:371–7. 29713009 10.1038/s41569-018-0004-1

[20] Assad F , El‐Sherif MMA . Effect of drinking saline water and feed shortage on adaptive responses of sheep and camels. Small Ruminant Res. 2002;45:279–90.

[21] Wang X , Hua T , Zhang C , Lang L , Wang H . Aeolian salts in Gobi deserts of the western region of Inner Mongolia: Gone with the dust aerosols. Atmos Res. 2012;118:1–9.

[22] Schmidt‐Nielsen K . The physiology of the camel. Sci Am. 1959;201:140–51. 14443122 10.1038/scientificamerican1259-140

[23] Chittavong M , Jansson A , Lindberg JE . Effects of high dietary sodium chloride content on performance and sodium and potassium balance in growing pigs. Trop Anim Health Prod. 2013;45:1477–83. 23456787 10.1007/s11250-013-0385-4

[24] Spörndly R . Fodertabeller för idisslare [Nutrition requirements for ruminants]. Uppsala, Sweden: Swedish University of Agricultural Science; 2003.

[25] Leitch I . The Feeding of Camels. In Imperial Bureau of Animal Nutrition Technical Communication, vol. 13. Aberdeen, UK: Rowett Institute; 1940.

[26] Cargill K , Sims‐Lucas S . Metabolic requirements of the nephron. Pediatr Nephrol. 2020;35:1–8. 30554363 10.1007/s00467-018-4157-2

[27] Hansen JT . Netter’s Anatomy Coloring Book. Philadelphia, USA: Saunders; 2009.

[28] Hill RW , Wyse GA , Anderson M . Animal Physiology. Sunderland, USA: Sinauer Associates; 2016.

[29] Ambros V . The functions of animal microRNAs. Nature. 2004;431:350–5. 15372042 10.1038/nature02871

[30] Bartel DP . MicroRNAs: genomics, biogenesis, mechanism, and function. Cell. 2004;116:281–97. 14744438 10.1016/s0092-8674(04)00045-5

[31] Dykes IM , Emanueli C . Transcriptional and post‐transcriptional gene regulation by long non‐coding RNA. GPB. 2017;15:177–86. 28529100 10.1016/j.gpb.2016.12.005 PMC5487525

[32] Tonouchi E , Gen Y , Muramatsu T , Hiramoto H , Tanimoto K , Inoue J , et al. miR‐3140 suppresses tumor cell growth by targeting BRD4 via its coding sequence and downregulates the BRD4‐NUT fusion oncoprotein. Sci Rep. 2018;8:4482. 29540837 10.1038/s41598-018-22767-y PMC5852021

[33] Biggar KK , Storey KB . Insight into post‐transcriptional gene regulation: stress‐responsive microRNAs and their role in the environmental stress survival of tolerant animals. J Exp Biol. 2015;218:1281–9. 25954040 10.1242/jeb.104828

[34] Cao Y , Zhang D , Zhou H . Key genes differential expressions and pathway involved in salt and water‐deprivation stresses for renal cortex in camel. BMC Mol Biol. 2019;20:11. 30961536 10.1186/s12867-019-0129-8 PMC6454748

[35] Zhang D , Pan J , Zhou H , Cao Y . Evidence from ileum and liver transcriptomes of resistance to high‐salt and water‐deprivation conditions in camel. Zoological Lett. 2020;6:8. 32518679 10.1186/s40851-020-00159-3 PMC7275387

[36] Konczal M , Koteja P , Stuglik MT , Radwan J , Babik W . Accuracy of allele frequency estimation using pooled RNA‐Seq. Mol Ecol Resour. 2014;14:381–92. 24119300 10.1111/1755-0998.12186

[37] Langmead B , Trapnell C , Pop M , Salzberg SL . Ultrafast and memory‐efficient alignment of short DNA sequences to the human genome. Genome Biol. 2009;10:R25. 19261174 10.1186/gb-2009-10-3-r25 PMC2690996

[38] Wen M , Shen Y , Shi S , Tang T . miREvo: an integrative microRNA evolutionary analysis platform for next‐generation sequencing experiments. BMC Bioinformatics. 2012;13:140. 22720726 10.1186/1471-2105-13-140 PMC3410788

[39] Friedländer MR , Mackowiak SD , Li N , Chen W , Rajewsky N . miRDeep2 accurately identifies known and hundreds of novel microRNA genes in seven animal clades. Nucleic Acids Res. 2012;40:37–52. 21911355 10.1093/nar/gkr688 PMC3245920

[40] Zhou L , Chen J , Li Z , Li X , Hu X , Huang Y , et al. Integrated profiling of microRNAs and mRNAs: microRNAs located on Xq27.3 associate with clear cell renal cell carcinoma. PLoS One. 2010;5:e15224. 21253009 10.1371/journal.pone.0015224 PMC3013074

[41] Wang L , Feng Z , Wang X , Wang X , Zhang X . DEGseq: an R package for identifying differentially expressed genes from RNA‐seq data. Bioinformatics. 2010;26:136–8. 19855105 10.1093/bioinformatics/btp612

[42] Kim D , Pertea G , Trapnell C , Pimentel H , Kelley R , Salzberg SL . TopHat2: accurate alignment of transcriptomes in the presence of insertions, deletions and gene fusions. Genome Biol. 2013;14:R36. 23618408 10.1186/gb-2013-14-4-r36 PMC4053844

[43] Trapnell C , Williams BA , Pertea G , Mortazavi A , Kwan G , van Baren MJ , et al. Transcript assembly and quantification by RNA‐Seq reveals unannotated transcripts and isoform switching during cell differentiation. Nat Biotechnol. 2010;28:511–5. 20436464 10.1038/nbt.1621 PMC3146043

[44] Sun L , Luo H , Bu D , Zhao G , Yu K , Zhang C , et al. Utilizing sequence intrinsic composition to classify protein‐coding and long non‐coding transcripts. Nucleic Acids Res. 2013;41:e166. 23892401 10.1093/nar/gkt646 PMC3783192

[45] Kong L , Zhang Y , Ye ZQ , Liu XQ , Zhao SQ , Wei L , et al. CPC: assess the protein‐coding potential of transcripts using sequence features and support vector machine. Nucleic Acids Res. 2007;35:W345–9. 17631615 10.1093/nar/gkm391 PMC1933232

[46] Punta M , Coggill PC , Eberhardt RY , Mistry J , Tate J , Boursnell C , et al. The Pfam protein families database. Nucleic Acids Res. 2012;40:D290–301. 22127870 10.1093/nar/gkr1065 PMC3245129

[47] Lin MF , Jungreis I , Kellis M . PhyloCSF: a comparative genomics method to distinguish protein coding and non‐coding regions. Bioinformatics. 2011;27:i275–82. 21685081 10.1093/bioinformatics/btr209 PMC3117341

[48] Ma L , Bajic VB , Zhang Z . On the classification of long non‐coding RNAs. RNA Biol. 2013;10:925–33. 23696037 10.4161/rna.24604 PMC4111732

[49] Young MD , Wakefield MJ , Smyth GK , Oshlack A . Gene ontology analysis for RNA‐seq: accounting for selection bias. Genome Biol. 2010;11:R14. 20132535 10.1186/gb-2010-11-2-r14 PMC2872874

[50] Mao X , Cai T , Olyarchuk JG , Wei L . Automated genome annotation and pathway identification using the KEGG Orthology (KO) as a controlled vocabulary. Bioinformatics. 2005;21:3787–93. 15817693 10.1093/bioinformatics/bti430

[51] Cil O , Esteva‐Font C , Tas ST , Su T , Lee S , Anderson MO , et al. Salt‐sparing diuretic action of a water‐soluble urea analog inhibitor of urea transporters UT‐A and UT‐B in rats. Kidney Int. 2015;88:311–20. 25993324 10.1038/ki.2015.138 PMC4523423

[52] Guo L , Meng J , Xuan C , Ge J , Sun W , O’Rourke ST , et al. High salt‐diet reduces SLC14A1 gene expression in the choroid plexus of Dahl salt sensitive rats. Biochem Biophys Res Commun. 2015;461:254–9. 25869070 10.1016/j.bbrc.2015.04.010 PMC4428960

[53] Hirunsatit R , George ED , Lipska BK , Elwafi HM , Sander L , Yrigollen CM , et al. Twenty‐one‐base‐pair insertion polymorphism creates an enhancer element and potentiates SLC6A1 GABA transporter promoter activity. Pharmacogenet Genomics. 2009;19:53–65. 19077666 10.1097/FPC.0b013e328318b21a PMC2791799

[54] Nayyar H , Kaur R , Kaur S , Singh R . γ‐Aminobutyric acid (GABA) imparts partial protection from heat stress injury to rice seedlings by improving leaf turgor and upregulating osmoprotectants and antioxidants. J Plant Growth Regul. 2014;33:408–19.

[55] Ren C , Zhang J , Yan W , Zhang Y , Chen X . RNA‐binding protein PCBP2 regulates p73 expression and p73‐dependent antioxidant defense. J Biol Chem. 2016;291:9629–37. 26907686 10.1074/jbc.M115.712125 PMC4850300

[56] Amery L , Sano H , Mannaerts GP , Snider J , Van Looy J , Fransen M , et al. Identification of PEX5p‐related novel peroxisome‐targeting signal 1 (PTS1)‐binding proteins in mammals. Biochem J. 2001;357:635–46. 11463335 10.1042/0264-6021:3570635 PMC1221994

[57] Alberts B , Johnson A , Lewis J , Raff M , Roberts K , Walter P . Molecular Biology of the Cell. New York, USA: Garland Science Press; 2002.

[58] Salmena L , Poliseno L , Tay Y , Kats L , Pandolfi PP . A ceRNA hypothesis: the Rosetta Stone of a hidden RNA language? Cell. 2011;146:353–8. 21802130 10.1016/j.cell.2011.07.014 PMC3235919

[59] Huang M , Zhong Z , Lv M , Shu J , Tian Q , Chen J . Comprehensive analysis of differentially expressed profiles of lncRNAs and circRNAs with associated co‐expression and ceRNA networks in bladder carcinoma. Oncotarget. 2016;7:47186–200. 27363013 10.18632/oncotarget.9706 PMC5216934

[60] Wu H , Guang X , Al‐Fageeh MB , Cao J , Pan S , Zhou H , et al. Camelid genomes reveal evolution and adaptation to desert environments. Nat Commun. 2014;5:5188. 25333821 10.1038/ncomms6188

[61] Timmer RT , Klein JD , Bagnasco SM , Doran JJ , Verlander JW , Gunn RB , et al. Localization of the urea transporter UT‐B protein in human and rat erythrocytes and tissues. Am J Physiol Cell Physiol. 2001;281:C1318–25. 11546670 10.1152/ajpcell.2001.281.4.C1318

[62] Yang B , Bankir L , Gillespie A , Epstein CJ , Verkman AS . Urea‐selective concentrating defect in transgenic mice lacking urea transporter UT‐B. J Biol Chem. 2002;277:10633–7. 11792714 10.1074/jbc.M200207200

[63] Yang B , Verkman AS . Analysis of double knockout mice lacking aquaporin‐1 and urea transporter UT‐B. Evidence for UT‐B‐facilitated water transport in erythrocytes. J Biol Chem. 2002;277:36782–6. 12133842 10.1074/jbc.M206948200

[64] Jin XT , Galvan A , Wichmann T , Smith Y . Localization and function of GABA transporters GAT‐1 and GAT‐3 in the basal ganglia. Front Syst Neurosci. 2011;5:63. 21847373 10.3389/fnsys.2011.00063 PMC3148782

[65] Wang Y , Gu W , Meng Y , Xie T , Li L , Li J , et al. γ‐Aminobutyric acid imparts partial protection from salt stress injury to maize seedlings by improving photosynthesis and upregulating osmoprotectants and antioxidants. Sci Rep. 2017;7:43609. 28272438 10.1038/srep43609 PMC5341084

[66] Schrader M , Fahimi HD . Peroxisomes and oxidative stress. Biochim Biophys Acta. 2006;1763:1755–66. 17034877 10.1016/j.bbamcr.2006.09.006

[67] Fransen M , Amery L , Hartig A , Brees C , Rabijns A , Mannaerts GP , et al. Comparison of the PTS1‐ and Rab8b‐binding properties of Pex5p and Pex5Rp/TRIP8b. Biochim Biophys Acta. 2008;1783:864–73. 18346465 10.1016/j.bbamcr.2008.02.013

[68] Kurtz TW , DiCarlo SE , Pravenec M , Morris RC . Changing views on the common physiologic abnormality that mediates salt sensitivity and initiation of salt‐induced hypertension: Japanese research underpinning the vasodysfunction theory of salt sensitivity. Hypertens Res. 2019;42:6–18. 30390036 10.1038/s41440-018-0122-5

