# Supplementary material for: Identification of sodium homeostasis genes in Camelus bactrianus by whole transcriptome sequencing
Source: FEBS Open Bio. 2022 Feb 22;12(4):864–76. doi: 10.1002/2211-5463.13380 (PMC8972041; doi:10.1002/2211-5463.13380)
Supplement: Supplementary file 1 — Fig. S1. ROS detection after sRNA‐MIX interference in renal medulla cells of camel by red fluorescent imaging DHE probe (100×). sRNA‐MIX indicates three classes of small RNAs (sSLC6A1, sPEX5L and sPCBP2) co‐interference against SLC6A1, PEX5L and PCBP2. Scale bar, 100 μm. Table S1. The details of six camels. Table S2. The sequences of fluorescent group labeled RNA probes. Table S3. The inserted SLC14A1 sequences in SLC14A1 luciferase reporter plasmids. Table S4. The inserted LNC003834 sequences in LNC003834S1 and LNC003834S2 plasmids. Table S5. The sequences of different short hairpin RNAs (shRNAs). Table S6. The sequences of different small RNAs (sRNAs). Table S7. The primer sequence of candidate genes of salt‐resistance. Table S8. The significantly differential mRNAs and lncRNAs in the renal medulla of camel under salt stress. Table S9. The significantly differential miRNAs in the renal medulla of camel under salt stress. Table S10. The sequence of novel lncRNA LNC003834 gene. Table S11. Plasma Na+ concentration. [file FEB4-12-864-s001.pdf]

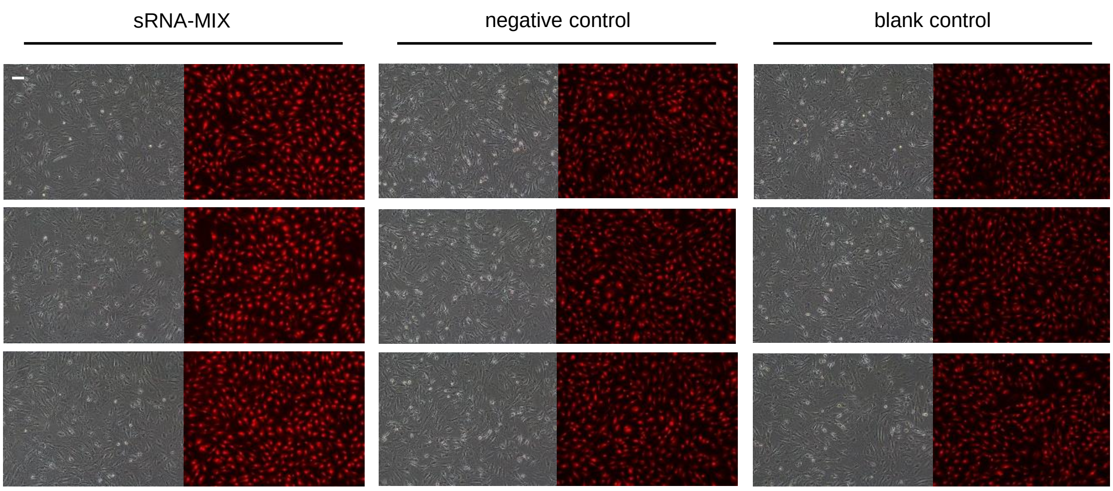

Fig. S1. ROS detection after sRNA-MIX interference in renal medulla cells of camel by red fluorescent imaging DHE probe (100×). sRNA-MIX indicates three classes of small RNAs (sSLC6A1, sPEX5L and sPCBP2) co-interference against *SLC6A1*, *PEX5L* and *PCBP2*. Scale bar, 100 μm.

Table S1. The details of six camels.

**Camel number**	**Group**	**Age (years)**	**Weight (kg)**	**Amount of xylazine (mg/kg)**	**Concentration of xylazine solution (mg/mL)**
1	salt stress	8	360.4	0.5	20
2	salt stress	8	347.2	0.5	20
3	salt stress	8	351.9	0.5	20
4	free diet	7	361.9	0.5	20
5	free diet	8	344.2	0.5	20
6	free diet	8	350.2	0.5	20

Table S2. The sequences of fluorescent group labeled RNA probes.

**RNA probe**	**Fluorescent group**	**Sequence**
lncRNA	FAM	5’-UCACUCAAGCUGGAAAUGGCUUGGGCGAAG-3’
miRNA	Cy3	5’-UGGCAGUGUCUUAGCUGGUUGU-3’

Table S3. The inserted SLC14A1 sequences in SLC14A1 luciferase reporter plasmids.

**Plasmid**	**Sequence**
SLC14A1 WT	5’-CAAGCGTTCCTCCTGGCTCTTGCCTGCGCTCTGTTCACTGCCTACCTTGGAGCCAGCATG-3’
SLC14A1 MUT	5’-CAAGCGTTCCTCCTGGCTCTACGGACGCGAGACAAGTGACGGAACCTTGGAGCCAGCATG-3’

Table S4. The inserted LNC003834 sequences in LNC003834S1 and LNC003834S2 plasmids.

**Plasmid**	**Sequence**
LNC003834S1 WT	5’-<br>TATTCCACCTCAGTGTGCTACATCAAAAGCTAATTTTTTATT<br>CTCCATCCTAAAATGTCCAGAAACATCCTTGTAGTCCTCTGT<br>AGGTTTGTTTTTTTTTTTTTAGGTTTTTCACATTCATCAGAAA<br>AGAGCATTTACAACCATACCTTTTGTCTTTGTAATATTTACC<br>ATTTATCAGAGCATAGCTTGATGCATTATTAATTTTAAAGAC<br>CTTCTCCTCAGGAGTAGAATGAAATAAACAAAAGGAGGAA<br>CATAGGGACACTGCCCTGTCATTCAGATGATTAGTGGGAAA<br>CCCTCCTCTCTTTATACTCTAAAGGTAGAGAAAATGAGCCG<br>AACATTAAATGTGTAAATCTGCACAGTTTTTTTTTCCTGGAA<br>TGGGCTGGACTCCATAGGCGAAAAATAAAATATGACTATAT<br>TTCATTACTTTCCAATGTACTGTCTCTGATTTTACTGTCACTA<br>CTCCTGAACATGTATCAGGAGGATTTCTATAGTTAGAAAAA-<br>3’
LNC003834S1 MUT	5’-<br>TATTCCACCTCAGTGTGCTACATCAAAAGCTAATTTTTTATT<br>CTCCATCCTAAAATGTCCAGAAACATCCTTGTAGTCCTCTGT<br>AGGTTTGTTTTTTTTTTTTTAGGTTTTTCACATTCATCAGAAA<br>AGAGCATTTACAACCATACCTTTTGTCTTTGTAATATTTACC<br>ATTTATCAGAGCATAGCTTGATGCATTATTAATTTTAAAGAC<br>CTTCTCCTCAGGAGTAGAATGAAATAAACAAAAGGTCCTTG<br>TATCCCTGTGACGGGTGTCATTCAGATGATTAGTGGGAAAC<br>CCTCCTCTCTTTATACTCTAAAGGTAGAGAAAATGAGCCGA<br>ACATTAAATGTGTAAATCTGCACAGTTTTTTTTTCCTGGAAT<br>GGGCTGGACTCCATAGGCGAAAAATAAAATATGACTATATT<br>TCATTACTTTCCAATGTACTGTCTCTGATTTTACTGTCACTAC<br>TCCTGAACATGTATCAGGAGGATTTCTATAGTTAGAAAAA-<br>3’
LNC003834S2 WT	5’-<br>AAGGAGCAGGGAAAGAATTCACCCTTCAAAGTTTTCTAGAG<br>TAACTGCTCTAGGAAAAGGGAAGGCAGGGACCTAGAGCGA<br>GGAAGGAGCCTGTCGCTTTAAGCGCAGTCAAGCTGAGGAGA<br>ACATTAAGGCAATCTTGGCCCTTCGTTATTTTTCACCCAGTG<br>AGCTAGTAATAAAACCTTAATTGTGACATAGGCCTCTGAAT<br>CCTTTTTCAGTGCTCACCCTTGGTGCTAACATTTGTCATTCTT<br>TTGCACTGCCCAACATATGAGCTCCATTTGTTGTTTTGACCA<br>TTCTGCACCTCCCATCATCATAAACGATAACACCAGTTCTTC<br>ACTCCCCGAACCTTCCGTGTTTGTACGACTTGGCAGACTCTG<br>AAGCCCGTACCAGTCAGATATGTCCAGCCCATGATCTAGAT<br>CACAACTTAGGTGAACGAGCATGGGATTATCATAACTCATC<br>TGAGGAAGCATGGCGGCGGGAGCCACGTGAGGCCGCAGGT<br>GGGT-3’

LNC003834S2 MUT	5'-AAGGAGCAGGGAAAGAATTCACCCTTCAAAGTTTTCTAGAGTAACTGCTCTAGGAAAAGGGAAGGCAGGGACCTAGAGCGAGGAAGGAGCCTGTCGCTTTAAGCGCAGTCAAGCTGAGGAGAACATTAAGGCAATCTTGGCCCTTCGTTATTTTTCACCCAGTGAGCTAGTAATAAAACCTTAATTGTGACATAGGCCTCTGAATCCTTTTTCAGTGCTCACCCTTGGTGCTAACATAACAGTAAGAAAACGTGACGGGAACATATGAGCTCCATTTGTTGTTTTGACCATTCTGCACCTCCCATCATCATAAACGATAACACCAGTTCTTCACTCCCCGAACCTTCCGTGTTTGTACGACTTGGCAGACTCTGAAGCCCGTACCAGTCAGATATGTCCAGCCCATGATCTAGATCACAACTTAGGTGAACGAGCATGGGATTATCATAACTCATCTGAGGAAGCATGGCGGCGGGAGCCACGTGAGGCCGCAGGTGGGT-3'

Table S5. The sequences of different short hairpin RNAs (shRNAs).

shRNA	Sequence
shSLC6A1	5'-GATCCGCTGCTCATGCTGGGCATTGATTCAAGAGATCAATGCCCAGCATGAGCAGCTTTTTTG-3'
shPEX5L	5'-GATCCGCAGCCCAACAACTTGAAAGCTTCAAGAGAGCTTTCAAGTTGTTGGGCTGCTTTTTTG-3'
shPCBP2	5'-GATCCGGAAAGGTGGTTGCAAGATCATTCAAGAGATGATCTTGCAACCACCTTTCCTTTTTTG-3'
NC	5'-CACCGTTCTCCGAACGTGTCACGTCAAGAGATTACGTGACACGTTCGGAGAATTTTTTG-3'

Table S6. The sequences of different small RNAs (sRNAs).

sRNA	Sequence
sSLC6A1	5'-GCTGCTCATGCTGGGCATTGA-3'
sPEX5L	5'-GCAGCCCAACAACTTGAAAGC-3'
sPCBP2	5'-GGAAAGGTGGTTGCAAGATCA-3'
NC	5'-UUCUCCGAACGUGUCACGUTT-3'<br>5'-ACGUGACACGUUCGGAGAATT-3'

Table S7. The primer sequence of candidate genes of salt-resistance.

Gene name	Primer name	Sequence (5' to 3')
SLC14A1	SLC14A1-F	GGATGCCGCTCAGTAAAGTC
	SLC14A1-R	GCCCTTCTTCTGTTTGCTCA
SLC6A1	SLC6A1-F	TTAGGCTGCCCGGCTTTAC
	SLC6A1-R	GACAGAGCCTCACCTTAACTTCCT

PEX5L	PEX5L-F	CTCACGGAATGTCAGTGGTTTAAT
	PEX5L-R	AAACACAGCCAACAGAAAATTCCT
PCBP2	PCBP2-F	AAACGATTTGATTGGCTGCAT
	PCBP2-R	TGACGGATCTCATTGATTTTGG
LNC003834	LNC003834-F	GAGGAACGTCCGAGCTGTTTT
	LNC003834-R	GCTGCTTTGCTGCTGTGAAA
miRNA-34a	miR-34a	TGGCAGTGTCTTAGCTGGTTGT
β-actin	actin-F	CCACCCTTCTCTTGACAAAACC
	actin-R	CCAAATAAAGCCATGCCAATC
U6	U6	TTCGTGAAGCGTTCCATATTTT

Table S8. The significantly differential mRNAs and lncRNAs in the renal medulla of camel under salt stress.

Gene	Salt_FPKM	Control_FPKM	log2(fold change)	pvalue
RNF38	2.60263	0.00487997	9.05889	0.0154735
PEX5L	1.19577	0.0063533	7.55622	0.0437093
PPP6R3	9.86102	0.429988	4.51937	0.0429138
SF3B1	48.0514	4.80974	3.32055	0.0445063
LOC105066559	37.2667	0.166626	7.80513	0.0249503
SLC14A1	228.139	20.687	3.46312	0.0336199
UTS2R	15.1762	0.19541	6.27916	0.0248486
IFNGR1	70.1743	3.72673	4.23496	0.0472229
PDE8B	55.2589	6.20641	3.15438	0.0464078
AKAP9	3.45717	0.00115313	11.5498	0.0365561
RSRC2	18.999	0.0210189	9.82002	0.0133057
PCBP2	6.95734	0.0149456	8.86267	0.0409414
MALRD1	12.0551	1.237	3.28473	0.040898
SLC6A1	1.01099	0.00616863	7.3566	0.0312548
HNRNPC	49.1538	2.35363	4.38434	0.0225014
LOC105072551	1.25831	21.9095	-4.12199	0.0430618
LOC105070098	0.825643	24.5895	-4.89638	0.0296767
TBC1D10A	0.0292026	16.0176	-9.09935	0.0243253
LTBP1	0.653411	14.2382	-4.44563	0.0362601
TCF20	0.0032182	2.0852	-9.33972	0.0336795
ARHGAP12	0.0063771	1.89662	-8.21631	0.0458438
ATP6V0A4	0.00868134	4.86342	-9.12984	0.0338363
LNC003347	6.06896	45.402	-2.90323	0.0471044
LNC003834	2.57553	0.231238	3.47742	0.046948

Table S9. The significantly differential miRNAs in the renal medulla of camel under salt stress.

miRNA	Salt_TPM	Control_TPM	log2(fold change)	pvalue
miRNA-148a	190250.4609	51526.4571	1.8845	0
miRNA-30a-5p	37002.06424	106960.9	-1.5314	0
miRNA-30e-5p	1301.323543	3561.126451	-1.4524	4.59E-280

miRNA-92a	2169.04152	4450.419458	-1.0369	2.82E-219
miRNA-450b	3510.025654	1409.13002	1.3167	1.61E-169
miRNA-455-5p	729.4761089	277.3744547	1.395	2.82E-40
miRNA-199c	73.23904654	304.716458	-2.0568	7.88E-39
miRNA-34a	361.7603423	735.5224847	-1.0237	4.81E-37
miRNA-486	107.0709244	297.8244654	-1.4759	1.57E-25
miRNA-29d-5p	361.0000754	128.9141559	1.4856	6.75E-23
miRNA-199b	58.16041931	169.4752269	-1.543	4.88E-16
miRNA-425-5p	182.5907717	60.44616426	1.5949	1.14E-13
miRNA-335	121.5159959	32.65222705	1.8959	4.52E-12
miRNA-379	294.2232977	139.9865374	1.0716	2.68E-11
miRNA-744	269.641334	129.5920568	1.0571	3.03E-10
miRNA-652	49.41734974	116.3729891	-1.2357	7.49E-09
miRNA-205	20.90734028	70.72766136	-1.7583	1.17E-08
miRNA-1	137.1014677	55.13594048	1.3142	4.50E-08
miRNA-141	155.9814296	67.45114031	1.2095	5.91E-08
miRNA-218	151.4198281	66.66025591	1.1837	1.62E-07
miRNA-136	33.95858905	3.728454992	3.1871	4.46E-07
miRNA-339b	26.35591986	71.06661181	-1.431	5.84E-07
novel 73	89.07794069	34.00802886	1.3892	3.75E-06
miRNA-409b	105.8038129	46.2102452	1.1951	1.00E-05
miRNA-499	100.6086556	45.98427823	1.1295	4.29E-05
miRNA-340	51.6981505	16.60857224	1.6382	5.45E-05
miRNA-2904	41.18112478	13.55801815	1.6028	0.00040076
miRNA-497	24.45525256	50.95555155	-1.0591	0.00060638
miRNA-184	4.054756902	17.39945663	-2.1014	0.0016126
novel 155	38.77361287	14.34890254	1.4341	0.0017203
miRNA-99a-3p	5.195157281	19.0942089	-1.8779	0.0020132

Table S10. The sequence of novel lncRNA LNC003834 gene.

**LNC003834 sequence (5’ to 3’)**
ttttATCACTAACAAAGGCTCTTGCACTTGTTTTTGTAAGAGTTTACAGGCATTTTGTCA<br>ATTTTCCAAGTCAAACCAACAATTAGAATACCTAAAGTAACAAAACAATAGGTACAA<br>TTTAATGAAGACCTAGTTGCCAGGAATTTTTCTAAAGTCCATACCACTGATATCAAGA<br>CTCCAAAAAGTGGACTAGGTTTATGACTTGATGGTTATACACAGATTGCACATTTCAT<br>TCTGTCTAATTTATACATGAATGGTGAAGTAggaaaattgagatttttctatACTTTACATAATTCAT<br>TTGATGCAATTAatttcaaaagctaaaataatgTATAGGAATCCCCTTGGACCCCATAAGATACTTT<br>TATCACTGAATGTGTGACCTATGATAGATCTGGTTTTCCATAAAAAAATTCATCAATCC<br>AAGGTAATAAAAACTGGGAAGCAGACATAATAATAATTGTGTGCTATTTAACTGCTCT<br>CGCTTATTTCctcttcactgttttcttttggtttttttgagaaTGAGTATGGAGAAATTGTGGCTATTGGTTA<br>GGGGGTCAATGTCAACATCCTGTTGCTAGGCTGACGTGTAGGAGACTGTATATGGGA<br>GAAGCAGGCTGACTCGAGAAATTGtgaacaaagccaaaaaaattacTATTCCAACTAAATATAAC<br>TGATTCAAATTATTCTTATCagttatatatattagttatagATTTACATATGACATTTACATAGATTTA<br>GAGAACTATTTTATCCTTTCATTCTTCTTGAAATTCTGATACAATGCATTTCTACCTTG

CTTTAAGTAAacctaaatttgttttccaggtTTTTTCAAACTAAACATACTGGTAAACCATCggttttta
gaaaattaaatggtGGATAAACATAGAATGTAAGACtaagaaataaacctttgaaGTCTAATTATATGATG
GaaatttgtatgttttctgaAAGCTTCTGTTCAGTATCTGCTGTACCATATTTGATGACAATAAACA
CCTTGGATTTCTAGAAAATGgcataaaatgtatattttcttttctacaataTAAAGGGCATTCTtagaattttccttc
tttcccattttattcctttaactTCTAACCAACCCCTAAAGTTTTTTCCagttgcttgtttatttgttgctGTATGTGT
TTGTGTTATTGTTTTTACTGTTCCTCTCATTAACTTTATTCCACCTCAGTGTGCTACATC
AAAAGCTAATTTTTTATTCTCCATCCTAAAATGTCCAGAAACATCCTTGTAGTCCTctgta
ggtttgttttttttttttaggtttttcaCATTCATCAGAAAAGAGCATTTACAACCATACCTTTTGTCTTT
GTAATATTTACCATTTATCAGAGCATAGCTTGatgcattattaattttaaagaccTTCTCCTCAGGAgt
agaatgaaataaacaaaaggagGAACATAGGGACACTGCCCTGTCATTCAGATGATTAGTGGGA
AACCCTCCTCTCTTTATACTCTAAAGGTAGAGAAAATGAGCCGAACATTAAATGTGTA
AATctgcacagtttttttttcctggaatggGCTGGACTCCATAGgcgaaaaataaaatatgactataTTTCATTACTT
TCCAATGTACTGTCTCTGATTTTACTGTCACTACTCCTGAACATGTATCAGGAGGATT
TCTatagttagaaaaataattgtgtttACTTATTAAAACATCCAGTCAAgcctaatatatattaaaaaaaaaaaagattc
agtctCATCTACAATGCcaatattttcccctttcctcaaCTCGGTTCTTTGCTTTGGTTCAGGAATTGTC
AGTGGGAAAGAAAGTGTGGTCCAGCCTGCAGCACTTCCCAGCCAGCCCTTCCGCCC
CACTTGTTGTTCTGGGGTCATGTCTATGAGGAGATGGAAGGGAAGGTGCTAGGCTAT
TTTATTTGCTTGGtggtgttttaattttatctgtgGAAGATATTTGCTGGCTGTTGTGCTCTCTGCAG
GTGTTGCAGACATTCAGTGTGATTTCTGTTTATGTTCACATTTCGGCTCTCTGCAGGG
ATGTACTGCCCCGTTCTCTCACTCGGGACGTGACCTCTTCCATTGTGACCTGTGATAC
CACCGTCCCGCTCACATTATGTTGTGTCGTCTGACAAACAGAACTTCTTAAGGCTGA
AGTTCCTCCACACTGGTAAGGCCCTCCTAGGTTGGGTCTCTTCACAGTGACTCAAGC
CATTTATTTCCATGGAGTTCACTTATTCCATAGAATGCTTAGTTCTGCTAACTGGGACG
TCAAAGAGCAGTTTTTTTCCAAATGACAATCAATCTCTTTATCCTCTGTTTTCATACTG
TTAAAGCATGCATTTAGTGTTCATATATTTTCACTTGATAGACAAGAACCGGAAGCTA
TGCTCATACATCTCTCAATTTCCAGGATACATATATCCCTCTGTACCAGAAAAGTCTCT
CACTACGtatggtaggctgaataatgatCCCCGGAAGGTATCTATGTCCTAATTCTCAGGCCTTGT
GAATGTGTTACATCATGTGTACATATGGAAAGACCTTGCATATGTGATTATATTTAGTA
TCTTAAAATGGAtagattatcctgggttatcgAGGCAGTCCCAAAATAATCGCCAAGGTTCATGT
AACAGGGAAGCAGAAAGGTAGAGAAGATGTGATGGGGAACAGAGATGGGAGCAAT
GTGGATGAGGAACGCAAGTGGCCTGCAGAAACTAAAAAAGGCAGGCTTTCCCGCTA
GAGCCGACAGAAGGAATGCAGCTCCAgggacaccttgattttagccctgcCCCAAGACCCATTTC
AGATTTCCAACCTCTAggactataagataataaatttgtgtggctttaagtcactaagttgTTAGTAATTTGTTGC
AGCAACAATCGAAAACAAATACACTAAACTGCAGTCAGGAAGTTCTAGGAAGTTGG
GTGCAGAGTTTCTGCAATGCAGCGATTCTGTAAGTCAAGAACGAGGTCCCAGCTTTC
CCTCCTGAGCGGCAGCTGTGTATCCAGAgtctggggagcagaggagaaaagTCAGTACACTCTC
ATAAAATGTATCAATGAATCTCCTCTTGAATTTCTGATCTTCTCTTAAAAAGTTTGGCA
GAGTTATAAAGATGAAGGTTCCAACGCCATCTATTCTGTCCCTTGGTAATTCCAGCGT
AGACGGGGGTAGCACTTCTCAGTAATCTTAGAATATGTGGAAATCATGTTCCCAGCA
GCAAATAAATAGCACATTTAACAGTTTTAAAGAAGAGTTTAATAATAGGATGGTTGAC
AAAGGTGTAGACAGAATGAAGAGAATCAGAGGACGGTGCAGGAACCTGGCTCCAA
CAACCttaagaaaagaggaggaagcgTTTATCAGAAACTAGAGAAGTTAACGGGGAGGCTGCT
TTGAGAGAGGTTGCGACCTATCCTTGTAGTTGCAAAGTGAACACACACCTCCCTCTC
TGATGGTTCCGTATCTGATGGTCCCCCACTGGCTTAAGCCAAACAGTAACCAGAGGG

CAAAGTCCAATGTGTTTCATGTAGGgtagaaagaaaactggagaaagatggagaaagttGGAGAGTGA
ATTTTGACGTGCAAATGAGAGGCATCTCATGCAGAGTCCTAATAAACCTGTTTTTCA
AGCTTTGTATCATCATCCAGAATTCTGGAGAATGGGAAAAACAAGGTTCAACAACAT
GTGACAAGTAGTTACACAAAATAGGAAATGtaagaatagaaatataaatgagtCACTAAGGAAAC
ATGGAGGGGAAATTGAACTCCATCTGGAGACTCAGAGTAGGTCCTTGCAGATCAGA
CAGCGTTTGTTGTTTGCAGTGATTACTAAGGATAAGTTGTACTTCACGTGGGTCATAA
GAAGGGGATGattttatagaagaaactttCTATTAGACCTCATgactgaaagagaaaactgaagtacagGAAAC
CTTGACTTTTTATTTCACTCCTGATATTTCTGGCTCCAAATAAAACTGTATCCTTTTTT
CACCCTTCTAAAGAGGAGAGACTGACTCTGCTGTTGCTGCTCAAATGCTGACCACTC
TGagcccccctctcccctcttcccctcttgcCCCACGTGTGGACGAGCCAATAATGCCTGCTTTGCTGC
CTCCCTTATTCCTGGTGAGAAAGTTCAAGCCATGGGGACTGCAGCCCACAAAACCTT
CAGCCTGCACGCCCTGCCCATCCACGAGAGAAACCAAAGCCCTTCGCCCAAGCCAT
TTCCAGCTTGAGTGActactctcttctcttccaaaggTCACAATATATCAGAGTTCTTTTCAGATCT
CATTAGTTTATGTGTGGTCTCATCACGTGGACACTCACATCAGTTACGGATGGAATAG
AGGTCACTCTTGTGCTCTGGGGACAACCAGCAAAACATAATCTAGTATAGAGAAGA
AATAACTTAAAACcttactttaaaaagataactgaTAAATTCTAATTAACTATGCCTCGATTAAAGA
AAATGACTAAATAATAATTGGCCCTTTGTTTGTTTATGAGATATACAAGAGACTTAAA
GACATTCTTTAACCAGTGTGCAGAGAATGGAAATTGTAATTCCATTTATTCCCTTTATA
TTTCCACATATCAAACGCTTAATGAGCCTTCTGTGTCTGGGATCTTGCCCTCGCACGA
GGCTATCCCAtccacttcctccccagcccttccttgtTCTGATATGATGTCAGGAATACTgtctttttatcttgtttt
caaTGGGGTGGTTAATAATTCAACTTTCTTTGCTAAGGAAAGAAATCCCTCTGCTAATT
GCTCACTTGTGTTATAATAAGGTCAGGCTCATTATAAAAGCAAACTTGATCCCTAAAT
GATATGCTGAAGCACGGGTAGAGAGTCAAGGACGAGGTTAGGATTTTTATATGTGTG
AATCTCTCTCTAAGACTAAAATGACCCGCTGTTTTGACAGCTACAAACTGAATcacaatat
tttcctttctccgAGTGGGAGTAGTCCTGGGCCATCTGCTTCCAAAGCTGCCTTCTTTCCCGG
CCTCTCACCCTTCCCGTACCAGCTTCTCCTTCCATCCGCTTTCGTGTTCCCTTTTGCT
GACTTCCCTCGCTCCGATGGTTGTACTCTAGCGGTCTGAAAGTCTGAGGGAAAATGA
TGCTTTGAACCTCTTGTTGCAGTGCCATTGGAAAATGATCACCTCAGTCACTAAACT
GCTTTTTAGGCCCAGGAACGTCATTgacccctttctctttttcttcgtttttctctgtgttctcacGTATAACGTG
GGAACAGTAGCCTTACTGTATCTCCAGCAGCAGACTCTTGTTTAGCGTCACTCGTCA
GTGTTATGATAGTGTTTTGAAGCCTTTACAGATCCGAAACTGTTGgtgttgatttttaattttctgttc
caCAAATGTGAACAGTGTATGTTATTTTCAGAGAGGATCTCTCatggtattaaaatttaaattactgcT
AGTTTTCCCACTCTTTCTTATAGTAGAGTATGATATTCAGTTCTCATCAAAGGCAAGTA
GTGAGACCTGAAAAACATTGTGAATTCATGTGAGAAAGTGGTCATCCTGTTTAACGG
GAGTACCAGAGACCATCTCCCAGAAGTCCCATTGCCCTGCGTATGGGTCAAATGTAG
AAATAAGACGATCAAGAAAATGTATTCCATTAAAGCCAAAGGAAGACAATTAAAACT
CGAGGTCAGTTATGTTTACACAGAAGCACATAGGATGTGAAGAAGAAGATTTCTGCA
ATAGGTGTTCAGatatacaaatatttgtgaatagTGGCAAAAGGCTTCTTAGAATTTTGGTTCAGT
ACTATGATTAAATggggaataaaaatggaaacaatctgtCCATTTGTTTATACTGAATTTAATATAAGA
GGGATTGTATTTAGAAAACccaacacatttaaaattgcaaatagCAATTCACAAGCTTTTAGGAGCG
TGCATCTCATCTCTGTTTTGTTAcaatatctaatatatatatatttaaatctactAGATTTTCCTGTTTGATA
AATTTTAGTGAACAAAAAGTTCACCGTATGAGATTTCCTTCAGATAACTAGAGATATC
TTAGTGGGTCCCAAAATAGGATTTAAGAAtgatactttataaaatatttaaagtctaatggtgaaattatttgtttctgt
tttctctcacaTCTTTTCATGAAGccaattgtttttgttttcttttcaaactcaTTTATTTGCCTTGAAACAACACT

TCTGAAGGTAGCCTTTCAGCAGTGGCTTAAAAAAGCATAATATCAGGAAGTTTAATG
ATTCAGCAGAGCTGAGAAACAGTCCATTAATTCTCACAAAAAAGCAAAACGTCAAC
AAACCTGAGCGAGGAAAGCTTTTCTTGTAGGGAGCTGTTAGAACTCCCTTTCCTGCC
AACGAACCTTTTCTAATTCATTGGTGGCTTTCTTAAATTAATCTGATACCGTGTGATGG
CAAAGAAGAACCACTACAGAATTACAGGTTCTAgaatattacatatatgtgtatgtaaatgtatggAATTT
TATCTTATTAATACAGACTATAAACTCAATGTACTGCCATCGCTTTTGTTTTATAGTCTT
TGTCATGAAACTGCCTTCTACTGGGATGAATGCTTCCTATATCTGGGCAGTTAAATAG
ATGGGCTGCTTTATCTTGACCTTTGGAGAATCTGTATTGAGTTGGGGGCTCATTTCAT
TGCTTTTACCATTTACAACTCTGTCTTCCAATGGCAAGggtgtttcaaatattttctcatcaaGCATGT
ACCTTTACTGAAAGGTCAGAATACTAAAATGCTATATCTCAGGGACTGAAATTATGTT
TCATCTCTTTCATGCAGGAAAGTCTAATTAGAAATGCACTACATGCGTTCCTTTGGGT
CCAAGCTGGCCGTGAACCTCACGGTGCTCAGTGAGGGCCCGAGCGCTCCCATGGAG
CGAGCACCCTGTTTTCTACAGCTTTGTTGACAATGTCATTCCTCCCAatattttgtacaattttttta
tGGACTTTGAAGTACGCTTTCAGTTTGTTCTTCGGAGGATCTTTACTGCAAGACCAAG
AAAGCAATACCGAAAGAGCATTATGCTATTTTGTGGGTCGACTTGGTAGCTCACTTC
CCCAGAGGAGTATGCCGTTCTACCATTTCACTCAATTCCgtaaaacagtaacaacaaaatcTGAA
CAATATACACGGTAGAAGTCAAGAAAACCAATTCCCTAGCCGTGCCAGGAGGTTGAT
CAAAACACAATCTTGGGGAAAGTTGggtaaaagaaacatttagaagGCATAATAACACAGGTgcta
aacagaacaaaaaacttGCTGTTTCTTGCAAGCATCAGGAAgtaatagatttatttatgtacttacaGCTTCTTA
CATTTTATGaacctcatttttctcactttagaTTGTTTTGGAGATTGTTTTACAGAGTGCATAtctgccaaat
tattttttatcatatcaTTTATTCATACACTATTTTCTTGTGTAGCTCTACTATAGTTTATTTAGCCT
TGCCTTACTGATGTTGTTTCAACCttttaatattactttaaaaaacagagtaaatgttgtgtatatgcgtgtgtgtacacgt
gtgtatacacacacgttTGCTTCCACATGCCTGTATTGTGTAAGCAAGTTTCCAGAAGTAGACT
TGCTATGTCAAGTGATTTcacaaatttttgaaataatgccAGATTTCCTTCTGATGATATTGAAACA
ATTTAGGTTTCAAATGATGATTAATGAGAGCCCATCTCCTGCACTTTGGATGCTAATG
TCTACTATTATCTGTTTGATCTTTGTCAGTATATTAGGAGAAAATTATTAGCACGCCATt
cattacacatacacacacgtacacccACATCCACTATTGtgcattttaggaaaacaaatttacCTTCTGATGTAAAA
CTATTCACTGCAGTATACCCTACCAGGTTATGCGGTTCCAGTTCTATTCATTAACTTTG
AGCTCTTGTGTAacttacaactttttaaaagttgaaggtAACTGAAAGATACGTAGATTCTGTTCTATC
AATGGTTTAATGACTTATCACGACCAAAAGTTGCTTTTATCTCAAAAATTCCTTTATCC
CATACTTACTTggcctctttttattttccctttgaacCAGTGAAAACATCTGCCTAGTTCATTCATATCA
CCGAGTAAAACAAATGTATTAATGTGGTTCATTTTTGTATCTACGTGTGTCATGATTTT
ATCTTcttatagaaattaaattttaaaagcatataggCATGCAGATATGCCATGCAGTGTATGATTGTATAA
TGTATCAGACATACACATCTTTATCTATGTACCTATTTATATAATTATGCATGTTTTCATC
ATTTGGGTCAAAGAAACATCACACTGGATGTCgttaaacaggcaaggaagactttaAGATTATGGC
ATGTGGAAAAAAGCTGTTGCATTCAGGGAGAGGGATTTAACGCATCTTCGTTGAAA
CAAAAGGTGAGTGTGGAGGCAGGTAGTGACAAACACTGCAGGGCAGACGTAGGGA
GGACTTGGTGTACATGGCTCTGCCCATGTGTGTTTGCTAATCGGTGCTTATTGAAGTT
AGGCTCCTACCCTCCCACATAGAGTCCCTAACTTTCCGGATTACTAAATTTCAAAGGA
ATGGCTCCCAGGACCCTGAGAAAGACATTCCTTAGATGTGAAACTACCAAGAGGCT
GGAAGAAGTTTTACATCAGAAAGGAGCAGGGAAAGAATTCACCCTTCAAAGTTTTC
TAGAGTAACTGCTCTAGGAAAAGGGAAGGCAGGGACCTAGAGCGAGGAAGGAGCC
TGTCGCTTTAAGCGCAGTCAAGCTGAGGAGAACATTAAGGCAATCTTGGCCCTTCGT
TATTTTTCACCCAGTGAGCTAGTAATAAAACCTTAATTGTGACATAGGCCTCTGAATC

CTTTTTCAGTGCTCACCCTTGGTGCTAACATTTGTCATTCTTTTGCACTGCCCAACAT
ATGAGCTCCATTTGTTGTTTTGACCATTCTGCACCTCCCATCATCATAAACGATAACA
CCAGTTCTTCACTCCCCGAACCTTCCGTGTTTGTACGACTTGGCAGACTCTGAAGCC
CGTACCAGTCAGATATGTCCAGCCCATGATCTAGATCACAACTTAGGTGAACGAGCA
TGGGATTATCATAACTCATCTGAGGAAGCATGGCGGCGGGAGCCACGTGAGGCCGC
AGGTGGGTACAGCAGCAGAAGTACTAGTGGAGACAGTTTACAGAATGTGGGAGAA
GCCGTCAGGAGTGAGTTCAAGGGTTATTTCGCATTATTGTAGCATTTTTCTTATGTGT
GTGACCCCCAGATTTGGTTCTTATATTCTTCTGAAAATTTGAGTCACTTACTCtcttttagatt
atttccatacCTGCTTAAATTAGCTAGAGtatgtttctaatatttaaaactaaaaagccAACACTGTTCTACTTA
GCAGTTGCTTCCACAATAATAAAAGTTTCACtcttcaagaaggaaaagaaaaaaaatctataaatccTCCC
TTATATATAGCTACCTTAagggaaataaatccacacacatgcattcttaaataatttttaaattttatttaacacattcagtttttgt
gattcttttacttttcagattgatttttttttataatgcttTTGTGGGTAAATCCTATAGTTACATTTActagagaaatagg
aaaatttcAGGGTGACTATTTGAATAGTCTGTATCTACTAGTCTATGAGTTTTTCCAACTTG
TtgcaatattttcaaaatacaaagaacacAAGATTTTTATTGGAAAGCAACATGGTTCACACTGCTGG
CTCTTGGCAGATGAATACTTCAAGTGGAAGCCTTTAAGCAAACTTCAAAGTGGTAAA
TATGAGCAGTCCTCCTGATGACTCTGTGTAAGTTACCTGCTTTCCTAAAAAGCCaaaga
gagactgaaagaaatgCACCTATTCCCTAGACTATTTGAAAGAATTGCACctattccttaattttatttaggT
TACCTAAGTAGCTGAGAGGCTTCAAAACACTGACCCATCTATATTACCTAGCTCGAA
CTCTGTGCCATTacaaaatgtttctctttgcagcatttgatttttgttgatttcttaaatttaaaaaaaatcagtgtaaaatatacataata
cagAATTTAGCATTTTAATCTAACTACAAGTGTATACAGCTCACTGGCATGAGTTACAT
TCACACTGGTGTATAACCGTTACTACCGTCCATTTCCAGAATATCTTTCATCCTGCAG
AACTGAAACCCTGCACCTGTTtaataataactccccattctctgcTCCACCTGgcccctggtaatcaccattctac
CTTTTTTTCTATGAATTGGACTACTCTAAGTAGCTCATGTACgtagaatcatgcagtatttgtcctttttg
actggcttctttaacttagcataGTGTCTTTAAGGTTCACTCATGTAGTAGCATgtgaatttcattccttttttaaagtc
gAATAATCTTGCACTCCATGTGTCGGCCAAGCTCTGTCCCGCTGTCTGTCAGTGCACA
CTTAGGCTCCTTCACCTTCGGTCAATTGTGAGTAGTGTTGCTATAAACATGGTTACGA
AGATGTCTGTTGACGTCCTTGGCCTTAATTACTTTGAGTCTATACCCAGAagtcagattgctg
gatcatatggtaattctattttcagctttttgaggGACCTCCCTACTGTCTTCCATACTGGTTGAGACATTTT
TTATATTCTCAGTACCAGTGTACGTgcgttccaatttctctacatccttaccGACACTTGTTATTACCAC
TTTTAAAACAATGGCCATTCTAATGAGCATTCGGTGTTCATCTCTCTGTAGTTTTGGC
TTGcgtttccctaataattagtgatgttgagcatcttttcatgtgctttggATTGTTTGTacaccttctttggagaaatgcttatTCAT
CTTTGATTGTCTTGTGTTGCATTTTGCTGTCGTCGTTCAGGTTTAGGAGTCCCTGTGT
ATGTGGGCACTAATCCCCATCTGATACACAGTTTACGGTATTTgcttccattctgtgggttgcctctT
TGCCTTGTTGATAGTCTTCTCTGCATACCAGTTTTGCGCTTTGATGAAGTCCCGTGTG
TCTGTTTCGTCTTtagttgcctgtgcttttggtgtcgtgTGCAAGAAATTATTGTCAGGACCAATGATG
TAATGAAGCCttccccttatgttttcttctaagagttgcATAGTTTCAGCTCCTCAAtttaggtctttggtccatttgaat
taattttggtGTATGGTGTTagataagggtccaacttcatccttttgcatgtagatatccagttttctccAGATCCATTTGTT
AAAATGACTGTTCTCTTTTTCAGGATTGGTTTGTCTATttggggtcccttgagattccatacaaattttaga
attggatttttctgtttctgcaaaaaCATCCTTGGGATTgtgatagggattgtattgacTGTGTAGGGTGCTTAGAG
CAGCGCTGACATCTAACAACATTGAGCTTCCAACCAGTGAACAGTGATGCCTTTCcctt
tgtgtcttcttcagtttcttccagcATCgtttacaattttcattttacaagtcTTTCAACACTCAAGTAAATTCCTAAG
TATGTTGTTCTTcctgatgctattgtaaatggaattgagAAGGGCATTTTAGTCAGCTTGAAGAGAGA
CTTTAACACTAATGGAAGCTTTGTGTTACTAAGATGTCATGAAATAAGTGGCACATTG
GAAATATAATTCAGATCCTCTGTTTTCAAGTCCAGCTCGTTGTCTGTTACCTCTTAGC

ACTGAAGCCtgaaatattcttaattttatatacaaGAATGTGGTACTTGAAATATTAGTCATAAGCATC
ATGAGGACACTGATGATGTAAAGTGCTCCGTAATTTTATCAGTTTGCTACAAAGAAA
CTTTATGAGAGAATTCAAATAATTACTAGACATAGTCAAAGTGTGGAAGCAGAGTTC
AAACAGAATAAATGACCTAGTCAAGtttattctgtaattttgaaACCAGAACAAATTAAGAAGCT
TAACAAATTAACTTTGTGAATGAACTCTAAGGCAGCATCTCAAAATGAAGGCAGAAT
TTGAAAGCCAAAGTCCAGGCTTTCTAAGTTGAAAGCATGATGTGTCTACTGAGAAA
ACCCAAATGGTTTCTGAGGGGTACAGTCAGGTATTATTGCATTCCTGGtttgagatttttattaatt
attgctGGAATGTGAACAATTAATGCTCCTGCAAGTTTGAAAGAACACTAcaatacaattaatttta
tttttctcaatactGAAAGTAGTAATATGCAGCTCACTATAGCATATTAGAAGTAAAAATCATTA
GTACCTTTGAGAACCTATTAGTGACTAATGAAATGTGTTTTACCAGTTATCCTTTCtgtaa
aaagaatattaattgtGATctatgaaattttcaaatatttttgtaactGATCATGTGCCATGTTTTAAATCATTTGGG
GAGAGTTTTTCTTAGTAAAGCTGGTTGGAAATTAAAATCATCCAAATTTTTGAATAGT
AATATGGCAATGAGTCAAGAGCCCCCATAAAATTCACAGAATATGACtaataatctgtaaaatga
aaaatttagaaataatagtGATTTAATGAATTGTCCTTTGAAAGAAACatactgacttctttttttataaaagcaaga
GTTGGAAACTCAAATTTTGAAATAGATTATTatgctattattaaaaacagtgctgctgaaaaaaaaaactgaca
ggGTTAATTGCTACAAgtaaaattcaagtaaaaaagaataaaacggTATATAAGCAGGTTATCCTATTTT
GTGAAATTAGCTAAAGCAAACACACCGTGTACTATGTACCCACAGAGGAACACACA
CCGGAGGGGACAGAGTTGAGGGAAACATTCAGAAATATAAATGCTGAGTAGCCTTG
CATATTAGGATTACAGgtcacaataatttttaaaataagtttccacgttttagaaatttattttaaaaagcacatcttATGTGA
AGATCTAAATTTTTTACGTTCTTTTTATAAGTGAATGCTTTATGTTTAACAGTAAATCA
TTCACATtttgtattagaaaataaaatcttgtcaCATATTATGTGTAGTCACCTTTAGAAGTTTTCACCA
TAAATGAATacagtgtatattttaaaactaaggaCACATTTTCTAACAATGTAAAACATAATAAGGCA
AAAATGTAACCCCTTTCCCCCAGCTAATTATTTCTGTTCTACCTGAGTGTTTTTCCTTA
AGGTTAGTTTTCTACATTGGCAGTGCCTGTGTTCAtactttttccctaaaaatataacttttaagaGATGA
GCAGGCATTGTGCTCTTATTTTTCATATCAAATACTCTTGGTGAGAGTTAATAATCCAC
CGACTGTGGTTTCTCTCTGTAGAGGACGTGTTGGGATTTCCCTGAAGGACGTTAACA
GTGGTCccatgattcaatatttttacctTGAGGTTTCCGTACCATCCACGCAGAGTGGAACTTCTG
AATATTTAGTAAGAAAAACTGATCAGCTGCAAACTGAAgtaagctttttcttcttttccctaaatagcttttt
aaaggttttattttttttataactgattATCTTTCCTCATGTCATATTCAAATTTAGAACCAATTTGAGAA
GAAAGGATTTCAGGCACTCCTGTGTACGGGGAGAGAGGAGATAGAAGAGAGATTAA
TTAGAAACAGTGAactgattccttaaaaaaaattggagtccTTTCTTAGATATTGTTGAATGAGGCAG
GCCCCAATGAGACGGAGTCTGCTACTCCAAAGGCAAACAGAAAATGGAATACTGAA
GAAATGATCACATGATGGAATTCTGAATTCACTTCCATCCTTTAACTTTCAGAGTACG
TGTCTGAAATGTTGTCATTGTGCTCAGACCAAAACACTTGACGAAGTCCTGGCGTGT
GAGAGCTGGACAGGAGCTCTGGCGCCTGTGCTTTTCTTTTAGATGTGGAAGCTGAG
GTTCCAGAAAGTGAAGTAACCTGCGTTCTTTAGCACTAgcattctaaaggaaaaaaaaaaaaagttgt
atttacaACTTAACTGTGAAGTTTAAATCTAGATTAGCTATTTATACACATGGTCATCGTTG
TCCCCAAGCAATTATATCTTCCTTGATGCAACTGAGATACAGACACTTACAGCAACAC
TCAGTCATTCACAGCTGAGCCCTCACTTGGCGTCCAGCTGAGAGGCAAAGCGTAAA
GGACGGCCTGCGCTTGGGAAGAGAAGCGCTAGTCTGAGCAGCTCCTGAGAGAACA
GATACGCAAACTGACACTTGGTCAAACCACACGTACGAAATAGAGCTCTGACCCGC
AGTCGGCAGCAACTGATATTAACAGCTGTGGACTGCAAataggagttcgggatttgtagatattaacta
ctacatataaaatagataaacaacaaggtcctactgtatagcatagggaactatcTTTAATACCTTGTATTGGCccagagtg
aaaaagaatatgaaaaggaaactgtatgcatgtatgtataaaagaatcactatgctgtacacattataaatcgactgtacttacatttaaaaagt

atttagagTTAATAAAGTTGTATTCACCCCAAAAATAGTCAGGACTTAGCCCATAATTACTc
gctttcctaatttttttttggcctttttccCATTTAGGACTAACCAGAGAAAGCCACAGAAGCTCTTCTAC
CCAACTGCACAGGATGTCCACTCTGtgctgtaGCTGGGAGCACTGTGTTGACACAACTC
AGTCTTATACAGAAGAGAGTAAAAGTGTTCTGCTTCATCCTTCCAGAGGCCGTTGTG
CAGAATACTGACAGTTTCCGCAAGAGGCAGTCATCGCAGTAAACACATACATTATGT
GGAGGAGGCTGCGTCTCTCCAAGGGAAATGGAGTCGGTGTATTGAGTCTCTAACCTT
CAGATCCTGAGTCTGAATGTATCTATGTTTCCAATGCAGTCGTCGACATGTGCTGGCT
ATTTCTTGGTGAGTAGGCCAGCAAATTCACCATGCCACTCCTTGagactctttttcttcctctcaggc
TTTCCCAGGGTATAAGCTGCGTCAGTTAACACTGGGTGCCATGCCGATGCTAGGAGT
CGTAGTAAAGAAATCAGCTTGCTGCAGTCATTAGTTAAGGACTTCAGAAGTGCCAAG
ACCCAGGAAACACGCGCATGCATCTGGACTGTGTGTCAGTATGTGCACTGATGTACT
GCCTGTCTAATCCTCACAGCTCAGTGAAAAGGTGGTGTTATCTCCACAgaacagatgggaaa
ctgaggcctgtaAATGcagagtaacttgcccaaggcaaCACGGGAAATAGAGAAGATGGATTTGAATGC
AGGGAGTCCGGTCCTATAGCCTATTCTGGTAGAGAGTTTCTGAAGGTTCATTAACTTT
TGGTATTTGTCCAGTTTGGGCGCCTTTACTCTCTGGTGACGTTGTCGGAGTGGAGTC
CTCGTTAGCAGGTGCTCGCGGTGGTCCTCCCATTCCCTTTTCGCCTCCATTGCTTGCT
GTGTCTCATTAATTTCTGACTCTAAGTGTCTCCCAAATTTGTCCCATCTTCCCCATCAC
ACCTACTGCTGCCTCAGTGCAGGCATCTTTGGAACATAACAGCAGCCTCCTCGTGTC
TTCCCTTTCTCCATTTTCAgtatcacccccacccccctgattATCCACACTGACAGTAAAGTGAATG
TTTTTTTTACAatgagtcagtttctggtgtacagcacaatgtcccagtcatgcgtatacatacatatattcgttttcataatctttttcatg
aaaggttttacaagagattgaatatagttccctgtgctctacagaggaaagtttttattacttatttttatatataatggctaacatttgccaATG
TCAAACGTccaaatttctcccttcccacccccctctttcccccataaccataagattgttgacTACGTGAAACCAAGCTT
ATCATGTCTTTCCCaggattaaatgtaaaataatatctATCATATGCATATATCACATGTTACTTACCC
ATTCAgcagttgatagacatttgggtcatttctacATTCTGGCTGTTAGGAATAGggttgttatgaacattcatgtgaaa
ggttttgtgtggacacacgttttcatttctcctggatgcatacctaggagtggagttgGTGGGTCCCATGTTTTTTTGAGG
AACGTCCGAGCTGTTTTCCAACGCAGCTGTCCCAAGTAACGTTTCACAGCAGCAAA
GCAGCGATGTATAAGGGTTCAAATCACCCCACGTTCTTGTTGCcaacatgttatttttctttttctttcttt
cctttttgacaCTAGCCAATGAAACTAACACTAATAGATATAAAGTTTTGTAAAGATTTGCA
TTTACACGATGgctaaagatgttgagcatctttttcggtgctttttggccatttgtgtatcttctttggagaaatgtctattcagatctcat
tttttaaactttgttattttttaatttgagttatatgagttctttctatattttagatacaagtccCCTATCAGATATAGGACttgcaaata
ttctttccttGCGTAGAactgtcttttccctttcttgatgGTATCTTCTGAAGCATAAAAGTTTGTAATTTTG
ATGAGTTTTTCTGTTGTCGTCGTATTGTAAACTGCTCAACCAAAGATTGTAAGGATTT
ACCCCTAAATATTCGTCTAAGAATTGTATAGTTTTAAACAAGGACATTTATTTAGCCCT
GTGAGCTATCTGGAGTTACTTTGGGGTGTCTGCTGTGAGGAGGGGGTCCAGCCTCAT
TCTGCTGCTGCAGCTGTCCAGCtgttccagcactatttatttgtttgaaagaCTCATTTTCCCTCCTGAG
TTATCTTGTTAcgttgattactatagctttgtagtaaggtGGGACCCTCCCGATTTTGTTCTTCTGTTGA
AAGATACTTTTAGATATTCtgggtcctttgcatttccatatgaattttaggatctgccaaacttctgaaaaaaatgcagCTG
TTATTTTGATGCAGATTGAATTAAAGATGTAGATCTATTTGGGAAGTATTACCATCTTA
ATATTAAGTCATCTAACTCATAAATGTGggatatctttctttttttcattttaaaattttatttatgtattttacaatatcatatt
ttttacattgacttatagttgatgtacagtattatatgttcCAGGTATCCAATATagcaattcacaatttttaaaggttacactccatttgt
AGTagttatgaaatattggctatagtcCCCATGGTGTACaacacatccttgtagcttattttatttatacctgatagtttgtacgTC
TCAGTCCTTCACCTccatcttccccctcccctctccctcttcccactggtaaccactatcCTCTTTCTTCTCTACAT
TTGTAAgactgcttcttttctgttacattcactagtttgttgcatttttttttttttagattccacatgtaagtgataccttATAGTATTTG
TCtatgtctgactgacttcacttagcataatgccccccATGTCCGTccacgctgtacaccagaaactaatacaacattttaaatcaact

gttcTTCAATTAAGAAGCAAGCAACCAAgcaaccaacccatttaaaatgcGGACAAAAGaattgaacaga
catttttctaaaagaggaaatgcaggtggccaacaggaacatgaaaagttgctcagcatcaGGGCAGGGGAGTATATACA
TgcgggtggggagggctatgtggggactctgtattttctgctcaattctgctgtgaacctgaaactgctctaaggAAATagtctattaag
aaaaaaagtacacggaagtattttattctttttcatgctatCATAAATAGACTTGTTTCCTTAGTACCATTTTCAG
GTTATTTATTGCAAGGATATAGAAATATACTTGATTTTTGTGTAATGATTTTATATCCTG
AACACTTCCTGAacttattgaattataattacttatatttttatagttaaatacATATGTTTTAACTAAATACTTCA
ATATTAGTATTAATTATAGTAGTTTTTAGTAGCTTTCTTAGACTTTATATATACAATCATG
TCCTCTGCAAACAGAGATaggtttccttcttcctttccaattgggctgcattttaattctttttcttgtatgaTTGCCCTGG
TTAGAACCACCAGTGTGATATTGAAAAAGAGTGACAAAAGGAAGCTCACTTTGTTG
CTGACCTTAGGCGGCAGCTTACAGTGTTTCACCATTAGGTATGCTCCTAGCTGTGGG
ATGTTCATAGAGCCCATTTATCCAGGTGGGGGAATTTTTAtcctattcctagtttgttgagtgttttttttgt
catgaaaCAGTATTggtttttgtcaaatgctttttccataTATATTGAGAGGATTATGAGTTTTGGGGGTTT
TAGTCTATTGATATTGGTATGTTacaatgatttttattatgttaaactGACTTGCATTCCGGGGGTAAAC
CCCACTTGGACAAGGTGTAATCCTTTTGCTATATTTCTGAATTTGGCTCGGTATTACAT
TTGCTGAGAATGTTTGTGTCTATATTTATCAGGGATGCTGGTCTGTGGCTTTCTTGGG
ATGCCTTTGcctctttgtactttttttttttttttgcaag

Table S11. Plasma Na^+ concentration.

**Condition**	**Plasma Na^+ concentration (mmol/L)**	**mean**	**SD**	**pvalue**
salt stress	150.55	151.9333333	4.0368593	0.2271583
	156.48			
	148.77			
free diet	148.37	147.99	2.5810657	
	150.36			
	145.24			
